# **Optimization Strategies of Na**_**3**_**V**_**2**_**(PO**_**4**_**)**_**3**_** Cathode** M**aterials for Sodium-Ion Batteries**

**DOI:** 10.1007/s40820-024-01526-x

**Published:** 2024-10-04

**Authors:** Jiawen Hu, Xinwei Li, Qianqian Liang, Li Xu, Changsheng Ding, Yu Liu, Yanfeng Gao

**Affiliations:** 1 School of Materials Science and Engineering, Shanghai University, Shanghai, 200444 People’s Republic of China; 2 Shanghai Institute of Ceramics, Chinese Academy of Sciences, Shanghai, 200050 People’s Republic of China

**Keywords:** Sodium-ion batteries, Na_3_V_2_(PO_4_)_3_, Cathode materials, Electrochemical performance, Optimization strategies

## Abstract

Na_3_V_2_(PO_4_)_3_ (NVP) has garnered great attentions as a prospective cathode material for sodium-ion batteries (SIBs) by virtue of its decent theoretical capacity, superior ion conductivity and high structural stability. However, the inherently poor electronic conductivity and sluggish sodium-ion diffusion kinetics of NVP material give rise to inferior rate performance and unsatisfactory energy density, which strictly confine its further application in SIBs. Thus, it is of significance to boost the sodium storage performance of NVP cathode material. Up to now, many methods have been developed to optimize the electrochemical performance of NVP cathode material. In this review, the latest advances in optimization strategies for improving the electrochemical performance of NVP cathode material are well summarized and discussed, including carbon coating or modification, foreign-ion doping or substitution and nanostructure and morphology design. The foreign-ion doping or substitution is highlighted, involving Na, V, and PO_4_^3−^ sites, which include single-site doping, multiple-site doping, single-ion doping, multiple-ion doping and so on. Furthermore, the challenges and prospects of high-performance NVP cathode material are also put forward. It is believed that this review can provide a useful reference for designing and developing high-performance NVP cathode material toward the large-scale application in SIBs.

## Highlights

- Optimization strategies for high-performance Na_3_V_2_(PO_4_)_3_ (NVP) cathode material are well summarized and discussed, including carbon coating or modification, foreign-ion doping or substitution and nanostructure and morphology design.
- The foreign-ion doping or substitution is highlighted, involving the Na, V, and PO_4_^3−^ sites, which include single-site doping, multiple-site doping, single-ion doping and multiple-ion doping.
- Challenges and future perspectives for high-performance NVP cathode material are presented.

## Graphical abstract

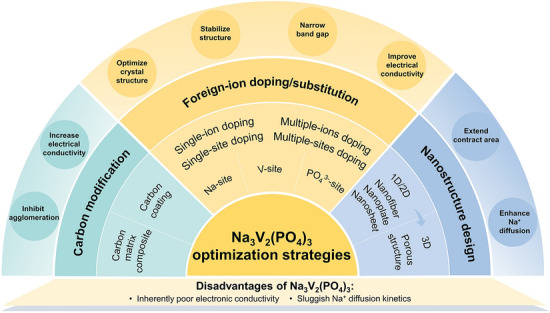

## Introduction

Lithium-ion batteries (LIBs) have been predominant in the energy storage due to their high energy density and energy conversion efficiency compared to other energy storage technologies. However, it is challenging to satisfy the escalating demand of markets for electric vehicles, portable electronic devices and energy storage systems at the same time, originating from the burgeoning consumption and volatile prices of lithium resources [1–4]. To tackle these issues, sodium-ion batteries (SIBs) have been widely proclaimed to be the up-and-coming candidates for grid-scale energy storage systems because of earth-abundant reserves and widespread distribution of sodium resources [5–8]. The energy storage mechanism and battery components are similar for LIBs and SIBs, which enables the fabrication of SIBs to accommodate for the available process equipment of LIBs. More importantly, the cathode and anode of SIBs can adopt cheaper aluminum foil as current collector without involving alloying reaction between Al and Na and overcharging phenomenon, thereby satisfying low-cost and high-safety requirements of energy storage systems [9, 10]. Nevertheless, there remain some challenges and inadequacies in the application of SIBs, including low energy density and unfavorable long-term cycling stability, which limit the popularization and practical application of SIBs [11]. The oversized radius of Na^+^ (1.02 nm) compared to Li^+^ (0.76 nm) generates larger resistance and volume change during the sodiation/de-sodiation processes, resulting in sluggish diffusion kinetics and diminished electrochemical performance. In addition, the higher redox potential of Na/Na^+^ (− 2.71 V) has an intrinsically negative effect on the energy density of SIBs compared with Li/Li^+^ (− 3.02 V) [12–14].

The electrode materials greatly affect the electrochemical performance of SIBs. Up to now, a myriad of researchers have endeavored to seek for appropriate electrode materials for high-performance SIBs [15–20]. The choice of cathode material has a significant effect on the improvement of energy density of SIBs. At present stage, the advanced cathode materials are mainly divided into three types: layered transition metal oxides [21, 22], Prussian blue analogs [23, 24] and polyanionic compounds [17]. Although layered metal oxides possess an admirable theoretical capacity of about 240 mAh g^−1^, the interlayer sliding and complex phase transitions during cycling process bring about severe lattice collapse and inferior cycling stability with rapid capacity decay [25]. Prussian blue analogs, with commercial advantages of low-cost and convenient synthesis, present excellent electrochemical properties. Unfortunately, they generally suffer from high interstitial water content (> 10%) within the framework, resulting in drastic structural collapse and capacity fading [26]. In contrast, polyanionic compounds with Na superionic conductor (NASICON) structure have been deemed as promising cathode materials owing to their robust and open three-dimensional (3D) skeletal structure, high ionic conductivity and good thermal stability [17, 27–30]. The NASICON-type materials as solid-state electrolytes were firstly proposed by Goodenough et al. [31]. Currently, numerous NASICON-type compounds have been reported widely and applied as electrodes or electrolytes for SIBs. The formula of NASICON-type materials can be represented as Na_*x*_M_2_(XO_4_)_3_, where x is the sodium content (*x* = 0 to 4), M is transition metal (M = V, Cr, Fe, Ti, Mn, etc.) and X is P, Si, Mo, etc. [32–34]. The stable structure of Na_x_M_2_(XO_4_)_3_ characterized by the strong binding energy of M–O chemical bond not only facilitates efficient migration of Na^+^ but also mitigates volume variation during the Na^+^ extraction and insertion processes, contributing to enhanced rate capability and cycling stability. Additionally, their flexible frameworks, with fine tunability, offer compatible structures and beneficial components meeting with multifunctional requirements of high-performance cathode materials [35].

As a typical NASICON-type cathode material, Na_3_V_2_(PO_4_)_3_ (NVP), with high structural stability and ionic conductivity, has received huge attention in a worldwide, which is similar to LiFePO_4_ cathode material for LIBs [36, 37]. NVP can deliver a high reversible capacity of about 117.6 mAh g^−1^ and a stable voltage platform of about 3.4 V, corresponding to a favorable energy density of about 400 Wh kg^−1^ in theory. On account of the tunable NASICON system in NVP, there exist ample ion channels that can be constructed for fast Na^+^ migration during high-rate cycling process, and thus NVP can obtain superior ion conductivity (10^–9^ to 10^–11^ cm^2^ s^−1^) [38, 39]. Furthermore, the high structural stability of NVP, only generating a minimal volume change (8.26%), can achieve long-term cycling stability in SIBs [39–41]. Unfortunately, NVP still confronts with two obstacles, intrinsically poor electronic conductivity and slow two-phase reaction (Na_3_V_2_(PO_4_)_3_ to Na_1_V_2_(PO_4_)_3_), leading to suboptimal rate performance and sluggish diffusion kinetics [42]. Therefore, it is urgent to optimize the overall electrochemical performance of NVP for fulfilling its commercial application in high-performance SIBs.

**Fig. 1:**
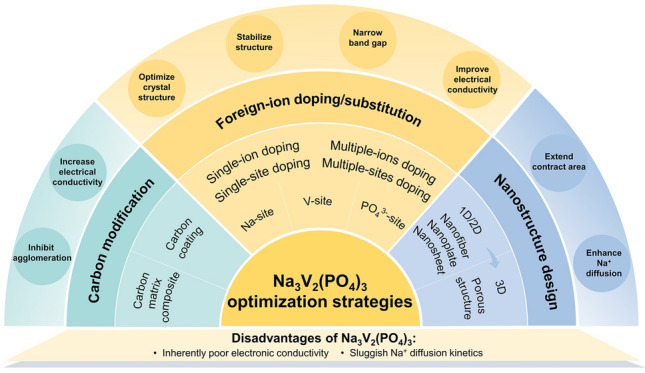
Outline of optimization strategies for improving the electrochemical performance of NVP cathode materials

Up to present, enormous efforts have been carried out on the optimization of NVP cathode material, and many optimization strategies have been proposed to boost the electrochemical performance of NVP cathode material. There are some reviews that have focused on the polyanion NVP material in the recent few years [43–46]. It is noteworthy that these reviews provide some information on the crystal structure, electronic structure, electrochemical performance, preparation methods and modification strategies of NVP electrode material. For instance, the review [43] mainly focused on the crystal structure and electronic structure of NVP and summarized partly the modification strategies, only including ion doping or substitution and carbon coating. However, there was no exhaustive elaboration on the ion doping or substitution. The review [44] summarized comprehensively the synthesis approaches of NVP material and introduced some modification strategies and practical application of NVP in full cell. But it seems to be lacking of excellent organization and detailed discussion for the optimization strategy of foreign-ion substitution. The review [45] only reviewed partial modification strategies of NVP including carbon coating, foreign-ion doping and construction of heterogeneous composite materials. However, the discussion on the modification strategies was not comprehensive. The review [46] introduced the progress of NASICON framework electrodes, including NASICON-type electrode materials, synthesis approaches, computational investigation and partial strategies for electronic conductivity enhancement. Nowadays, the reviews on detailed and critical optimization strategies based on the latest progress of NVP are still scarce, especially systematical and comprehensive discussion. Hence, in this review, we will summarize the latest advances in the optimization strategies for improving the electrochemical performance of NVP cathode material in a fresh perspective, and clarify their effects on the sodium storage performance of NVP, which are of great significance to further develop high-performance NASICON-type cathode materials for SIBs. Three kinds of optimization strategies are mainly discussed, including carbon coating or modification, foreign-ion doping or substitution and nanostructure and morphology design, as shown in Fig. 1. Among them, the foreign-ion doping or substitution is highlighted, which involves the replacement in the Na, V and PO_4_^3−^ sites, including single-site doping, multiple-site doping, single-ion doping, multiple-ion doping and so on. Furthermore, this review provides the prospects and challenges of NVP cathode material. It is believed that this review will be beneficial to guiding the design of high-performance NVP cathode material and promoting large-scale applications of NVP cathodes in the near future.

## Structure and Electrochemical Performance of Na_3_V_2_(PO_4_)_3_

### Crystal Structure

**Fig. 2:**
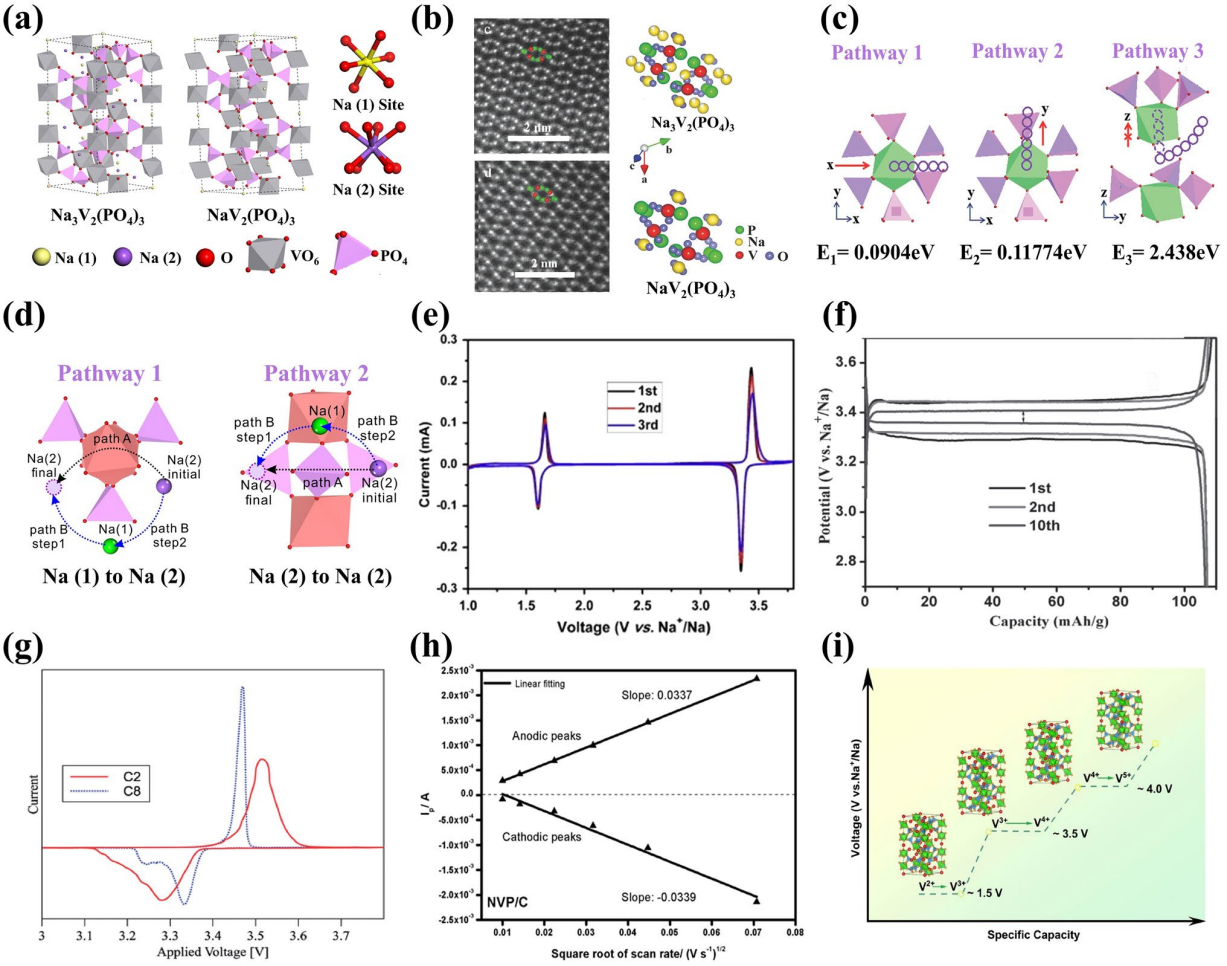
**a** Crystal structures of Na_3_V_2_(PO_4_)_3_ material. Reproduced with permission. [47] Copyright 2017, Elsevier. **b** STEM-HAADF images of Na_3_V_2_(PO_4_)_3_ and NaV_2_(PO_4_)_3_ along the [$1\overline{1 }\overline{1 }$] projection. Reproduced with permission. [35] Copyright 2014, John Wiley and Sons. **c** Possible migration pathways of Na^+^ in NVP lattice. Reproduced with permission. [49] Copyright 2014, Royal Society of Chemistry. **d** Sketch map of the direct diffusion route (path A) and stepwise ion-exchange route (path B) for Na migration. Reproduced with permission. [50] Copyright 2018, American Chemical Society. **e** CV curves of Na_3_V_2_(PO_4_)_3_/C. Reproduced with permission. [51] Copyright 2011, Elsevier. **f** Charge–discharge curves of Na_3_V_2_(PO_4_)_3_. Reproduced with permission. [40] Copyright 2013, Wiley‐VCH. **g** CV curves of C2 (carbon content 1.76%) and C8 (carbon content 6.95%). Reproduced with permission. [57] Copyright 2016, Royal Society of Chemistry. **h** Relationship between *I*_*p*_ and *v*^1/2^ for NVP/C. Reproduced with permission. [54] Copyright 2021, Author(s). **i** Schematic illustration of multiple V^2+^/V^3+^/V^4+^/V^5+^ redox reactions in NVP cathode. Reproduced with permission. [53] Copyright 2022, Elsevier

NVP possesses a rhombohedral lattice system belonging to R3 $\overline{c }$ space group (the hexagonal cell parameter: *a* = *b* = 8.74 Å and *c* = 21.84 Å), which is in good agreement with the first-principle calculation [46]. As shown in Fig. 2a, in the crystal structure of NVP, two octahedral VO_6_ are connected to three tetrahedral PO_4_ mutually to form a rigid 3D open skeleton (V_2_PO_4_)_3_ resembling “lantern” unit which interlinks each other along the c-axis. Additionally, there are two different interstitial spaces for Na ion sites located at independent oxygen environments: Na1 site has sixfold coordination (CN = 6) at 6*b* sites along the c-axis and Na2 site has eightfold coordination (CN = 8) at 18e sites along the b-axis, in which the occupancy rates of Na atoms are 0.843 and 0.719, respectively [47]. The Na ions occupied at Na2 sites are more susceptible to be extracted during the electrochemical reaction process because of the relatively smaller Na2-O bond compared to the Na1-O bond [43]. More importantly, the high corner-sharing structure with large interstitial spaces can provide a great number of Na ion channels and generate a low volume change (8.26%) for achieving high ionic conductivity and stable reversible cycle life. Furthermore, the high-angle annular dark field (HAADF)-scanning transmission electron microscopy (STEM) images of NaV_2_(PO_4_)_3_ and Na_3_V_2_(PO_4_)_3_ along [$1\overline{1 }\overline{1 }$] are displayed in Fig. 2b [35]. It is discernible that the phosphorus atoms (green spheres) and vanadium atoms (red spheres) in NaV_2_(PO_4_)_3_ and Na_3_V_2_(PO_4_)_3_ frameworks still remain well consistency with the same structure after Na ion's extraction, demonstrating a highly reversible stability during the process of two-phase reaction of Na ions in the NASICON skeleton [48].

The migration mechanism and pathway of Na ions have been identified in the NASICON-type Na_3_V_2_(PO_4_)_3_ structure with the help of first-principles computations, including three types of channels: pathway1, pathway2 and pathway3 [49]. As depicted in Fig. 2c, there exist pathway1 along the x direction and pathway2 along the y direction, corresponding to similar and favored migration energies of 0.0904 and 0.11774 eV, respectively. It is not feasible for Na ion's migrating along the z direction owing to the high energy barrier (over 200 eV). Thus, the pathway3 will undergo a curved course, which will bypass the octahedron VO_6_ and move into the gap between the adjacent tetrahedral PO_4_ and octahedral VO_6_, attributing to the possible activation energy of 2.438 eV. Moreover, the migration pathways of Na ions have been investigated not only at Na2 sites but also at Na1 sites, where Na ions are mobile in NVP framework. Wang et al. [50] proposed that the Na ions at Na1 sites would also engage in migration progress during the intercalation and deintercalation processes. The periodic density functional theory (DFT) simulations revealed three types of sodium-ion transportation routes in the NVP lattice. The first way of Na ion migration was directly from Na1 site to Na2 site with a highly activation barrier of 36.1 kcal mol^−1^, which was unfavorable. As shown in Fig. 2d, there remained two kinds of similar ion-diffusion ways focused on the Na2 site to adjacent Na2 site. The path A followed a direct course with high energy barrier of 63.22 kcal mol^−1^ and the path B was divided into two sequential steps, which formed a Na1 vacancy in an intermediate state. Compared with path A, the path B, named as concerted ion-exchange route, had lower activation barrier of 14.0 kcal mol^−1^ and thermodynamic energy. Therefore, it is significant to understand the electrochemical performance of Na_3_V_2_(PO_4_)_3_ cathode based on the structure and migration behavior of Na ions in the NVP framework.

### Electrochemical Performance

The electrochemical performance of NVP material mainly registers as its capacity, cycling stability and rate capability. In the cyclic voltammetry (CV) curves of NVP material, as displayed in Fig. 2e, there were two redox couples corresponding to V^3+^/V^4+^ and V^2+^/V^3+^ redox peaks at 3.4 and 1.6 V [51], respectively. In the high voltage of 3.4 V, two Na ions located at Na2 sites simultaneously involved in the reversible deintercalation and intercalation processes accompanied by two-phase transition reaction between Na_3_V_2_(PO_4_)_3_ and NaV_2_(PO_4_)_3_, providing a theoretical capacity of 117.6 mAh g^−1^. In fact, it is a difficult thing to achieve the theoretical capacity for pure NVP cathode material (Fig. 2f) [40, 52, 53]. For example, Dong et al. [54] reported that the NVP/C material only presented an initial capacity of 72.5 mAh g^−1^ at 1 C and lower capacity of 65.6 mAh g^−1^ after 100 cycles. For the relatively lower voltage platform, one additional Na ion would be intercalated into the Na2 site, providing 58.8 mAh g^−1^ capacity [55]. Consequently, it plays a vital role for NVP to be reasonably applied as cathode or anode with the electrochemical reactions as follows [56]:

1 $${\text{Anode reaction: Na}}_{3} {\text{V}}_{2}^{{3 + }} ({\text{PO}}_{4} )_{3} + {\text{ xNa}}^{ + } + {\text{xe}}^{ - } \leftrightarrow {\text{Na}}_{{3 + {\text{x}}}} {\text{V}}_{{2 - {\text{x}}}}^{{3 + }} {\text{V}}_{{\text{x}}}^{{2 + }} ({\text{PO}}_{4} )_{3}$$

2 $${\text{Cathode reaction: Na}}_{3} {\text{V}}_{2}^{{3 + }} ({\text{PO}}_{4} )_{3} \leftrightarrow {\text{Na}}_{{3 - {\text{x}}}} {\text{V}}_{{2 - {\text{x}}}}^{{3 + }} {\text{V}}_{{\text{x}}}^{{4 + }} ({\text{PO}}_{4} )_{3} + {\text{xNa}}^{ + } + {\text{xe}}^{ - }$$

3 $${\text{Overall reaction: Na}}_{3} {\text{V}}_{2}^{{3 + }} ({\text{PO}}_{4} )_{3} \leftrightarrow {\text{Na}}_{{3 - {\text{x}}}} {\text{V}}_{{2 - {\text{x}}}}^{{3 + }} {\text{V}}_{{\text{x}}}^{{4 + }} ({\text{PO}}_{4} )_{3} + {\text{Na}}_{{3 + {\text{x}}}} {\text{V}}_{{2 - {\text{x}}}}^{{3 + }} {\text{V}}_{{\text{x}}}^{{2 + }} ({\text{PO}}_{4} )_{3}$$

Additionally, some studies indicated that the additional reduction peak at about 3.2 V would also occur owing to the arrangement of Na ions by migrating from Na1 site to Na2 site in the local redox environment (Fig. 2g), while the peak disappeared fully after several cycles [57]. It is an amazing phenomenon that the appearance and disappearance of reduction peak at about 3.2 V did not affect the capacity of NVP [58]. Diffusion coefficient of Na^+^ (D_Na+_) of the pristine NVP can be calculated from the CV curves at different scan rates. An extremely low D_Na+_ of 4.05 × 10^–11^ cm^2^ s^−1^ for the anodic reaction and 4.10 × 10^–11^ cm^2^ s^−1^ for the cathodic reaction were obtained during the charge and discharge processes (Fig. 2h) [54], suggesting a sluggish kinetic characteristic of Na ion migration in the NVP. Thus, the pristine NVP cathode material exhibited low rate capability and poor cycling stability.

Furthermore, it has been a popular avenue to oxide V^4+^ to V^5+^ reversibly accompanied by more than two Na ions at a higher operating potential, resulting in improved specific capacity and energy density of NVP cathode (Fig. 2i) [53, 59, 60]. However, the electrochemical performance of pure NVP remains inadequate, which is attributed to two reasons briefly: (1) the weak intrinsic electronic conductivity with a wide bandgap, leading to poor rate performance and smaller specific capacity [61]; and (2) the larger size of Na ions impeding ion's transport, causing sluggish ion-diffusion kinetics and inferior cycling stability. Therefore, it is highly worthy of research to improve the overall electrochemical performance of NVP for achieving the desirable application in SIBs.

## Optimization Strategies of Na_3_V_2_(PO_4_)_3_ Material

To enhance the electrochemical performance, numerous optimization strategies have been proposed and applied to NVP cathode material for the promotion of the overall conductivity and ion-diffusion ability [62–65]. In this section, the optimization strategies of NVP cathode material can be summarized into three aspects: (1) carbon coating or modification, (2) foreign-ion doping or substitution and (3) nanostructure and morphology design.

### Carbon Coating or Modification

The coating or modification of carbon materials, which can boost the extrinsic conductivity dramatically, is an effective optimization strategy to improve the electrochemical properties of NVP [66]. Generally, the coating or modification of carbon materials can be divided into two methods, carbon coating and carbon matrix compositing, based on the current research progress and development trends [67–70]. Most of carbon coating or modification commonly contains high carbon loading content for preparing NVP/C composites, while the low carbon loading content with an appropriate thickness of carbon coating layer keeps challenging and also plays an amazing part in boosting the apparent electrical conductivity and electrochemical activity of NVP material. Table 1 gives the comparison of electrochemical performance of some reported NVP/C cathode materials based on the carbon coating or modification strategies.

**Table 1:** Electrochemical performance of some published carbon-modified NVP cathode materials

Cathode material	Carbon source	Discharge capacity (mAh g^−1^)/Rate	Capacity retention/Cycles/Rate	Refs
Na_3_V_2_(PO_4_)_3_@C	Critic acid	99, 1 C	94%, 1400, 10 C	[58]
Na_3_V_2_(PO_4_)_3_/C-GA	Critic acid and graphene	104, 0.2 C	95.2%, 250, 0.1 C	[84]
Na_3_V_2_(PO_4_)_3_/C	Ethylene glycol	118, 0.2 C	81.5%, 1000, 10 C	[68]
Na_3_V_2_(PO_4_)_3_/C	Glucose	124.1, 0.1 C	83.9%, 10,000, 20 C	[81]
Na_3_V_2_(PO_4_)_3_/C	Oxalic acid	115.1, 0.1 C	91.3%, 500, 1 C	[87]
Na_3_V_2_(PO_4_)_3_/N-doped carbon	Critic acid	114.2, 0.2 C	92.1%, 400, 1 C	[69]
Na_3_V_2_(PO_4_)_3_/N-doped carbon	Critic acid and CNTs	106.5, 0.1 C	98.9%, 2000, 20 C	[42]
Na_3_V_2_(PO_4_)_3_/N,S-doped carbon	Critic acid	123.19, 0.1 C	99.62%, 250, 1 C	[93]
Na_3_V_2_(PO_4_)_3_/N,B-doped carbon	Oxalic acid	114, 1 C	–	[89]
Na_3_V_2_(PO_4_)_3_/C@CNTs	Critic acid and CNT	98.7, 0.1 C	81%, 500, 2 C	[54]
Na_3_V_2_(PO_4_)_3_@C@CNTs	Ethylene glycol and CNT	112, 1 C	95.89%, 20,000, 200 C	[70]
Na_3_V_2_(PO_4_)_3_/rGO	Reduced GO	116, 1 C	90.1%, 400, 10 C	[75]
Na_3_V_2_(PO_4_)_3_/C-Mes	Critic acid	123.19, 0.2 C	90.48%, 5000, 20 C	[65]
Na_3_V_2_(PO_4_)_3_/carbon dot	Oxalic acid	112.3, 0.5 C	98.4%, 20,000, 200 C	[96]

#### Carbon Coating

**Fig. 3:**
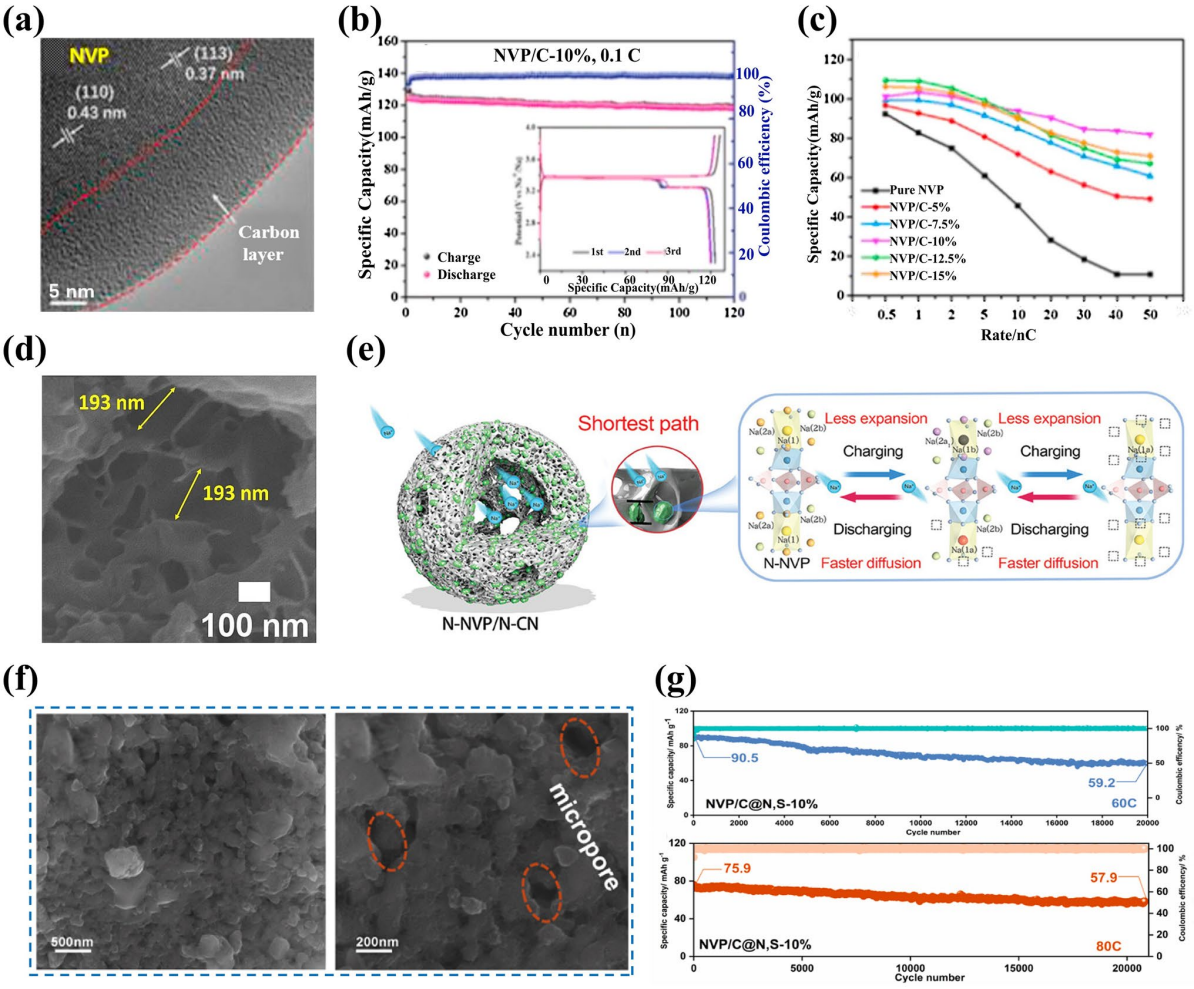
**a** HRTEM (high-resolution transmission electron microscopy) image and **b** cycling performance of NVP/C-10%, **c** rate performances of NVP/C materials with different carbon contents. Reproduced with permission [81]. Copyright 2021, Elsevier. **d** SEM (scanning electron microscopy) image of NVP@C at 950 °C. Reproduced with permission [58]. Copyright 2023, Elsevier. **e** Schematic illustration of nanocage structure and surface crystal modification of N-NVP/N-CN for fast reversible three-phase reaction. Reproduced with permission [92]. Copyright 2023, Wiley. **f** SEM images and **g** cycling performance of NVP/C@N,S-10% material. Reproduced with permission. [93] Copyright 2023, Elsevier

The carbon coating has been extensively used to polish up the sodium storage performance of electrode materials owing to its facile preparing process and effective functions. The carbon coating on NVP can be achieved by solid-state reaction [71, 72], sol–gel method [73, 74], spray-drying method [75], electrospinning [76, 77] and other preparation methods [78–80]. The conductive carbon coating on the surface of NVP not only increases the electronic conductivity but also reduces the side reactions between NVP and electrolyte, as shown in Fig. 3a [81]. In general, the function of carbon coating layer is inevitably influenced by two factors. One is the thickness of carbon coating layer, which plays a critical role in affecting the electrochemical properties of NVP cathode and serves as a buffer layer to alleviate the volume change during the charge and discharge processes. Specifically, too thick carbon coating layer may impede the Na^+^ mobility resulting in poor electrical conductivity [82], while too thin carbon coating layer cannot protect the crystal structure of NVP leading to unreliable structure stability during the electrochemical reaction process. The other one is the weight fraction of carbon layer, which exists an appropriate value because the high carbon content may impair the electrochemical activity of NVP [80]. Shen et al. [81] discussed the influence of different carbon contents (5, 7.5, 10, 12.5 and 15 wt%) on carbon-coated NVP materials, and found that the NVP/C nanocomposites with 10 wt% carbon showed a splendid enhancement of reversibility of Na^+^ insertion/extraction, exhibiting 124.1 mAh g^−1^ discharge capacity at 0.1 C with 95% capacity retention after 120 cycles (Fig. 3b). Noticeably, the NVP/C-10% cathode revealed the best rate capability compared to other samples (Fig. 3c). Additionally, the conventional carbon sources for carbon coating are mainly commercially available organic compounds, such as citric acid [58, 83–85], glucose [68], polyethylene glycol [86] and oxalic acid [87, 88]. Kate et al. [58] constructed a graphitic and porous nanostructured carbon layer on NVP using citric acid as carbon source by sol–gel method. The NVP/C revealed many beneficial pores attributed to the thermal pyrolysis of citric acid at high temperature of 900 °C, and these pores increased the contact area of electrode and electrolyte and offered more active sites (Fig. 3d), boosting diffusion kinetics with rapid ion-transport rate.

Furthermore, it is widely proclaimed that the heteroatoms (including N, S, B, P) doped into carbon coating layer of NVP/C, taking more extrinsic defects and active sites, can profoundly improve the electronic conductivity and facilitate sodium-ion transfer compared with the bare carbon coating layer [89]. Currently, nitrogen is one of the most extensive dopants due to more active sites scattered in carbon substrate leading to outstanding electrochemical performance of carbon coating layer [69, 90, 91], and nitrogen could be randomly doped in carbon layer and the surface of NVP crystal structure (Fig. 3e) [92]. Zhou et al. [93] synthesized N/S co-doped carbon skeleton enwrapped NVP/C@N,S-x% (*x* = 5, 10, 15) nanoparticles using thiourea as nitrogen and sulfur sources. The synergistic effect of N and S elements had contribution to providing abundant defects and active sites in the carbon layer which produced ample Na ion-diffusion channels and boosted ionic conductivity. Benefiting from the pyrolysis of thiourea, coral-like morphology was formed and the specific micropores architectures were constructed for NVP/C@N,S-10%, providing rich conductive networks and mitigating volume expansion to enhance its structural stability (Fig. 3f). Consequently, the optimized NVP/C@N,S-10% material realized excellent sodium storage performance, exhibiting a desirable capacity of 123.19 mAh g^−1^ at 0.1 C and an eminent discharge capacity of 90.5 mAh g^−1^ at 60 C maintained to 59.2 mAh g^−1^ after 20,000 cycles (Fig. 3g).

#### Carbon Matrix Compositing

Compositing NVP with carbon nanotubes (CNTs) [54, 70], reduce graphene oxide (rGO) [94], mesoporous carbon (MC) [95] or carbon dots (CDs) [96] has drawn significant attention from researchers in the field of optimizing electrode materials. These various carbon matrices are gifted with peculiar structures and high conductivity, which are beneficial to constructing high-performance NVP materials for SIBs [97]. Furthermore, the special structures of carbon matrices can effectively regulate the growth process of NVP grains and block the agglomeration of NVP particles, in favor of improving the crystallinity of NVP material and facilitating its electrochemical reactions effectively.

**Fig. 4:**
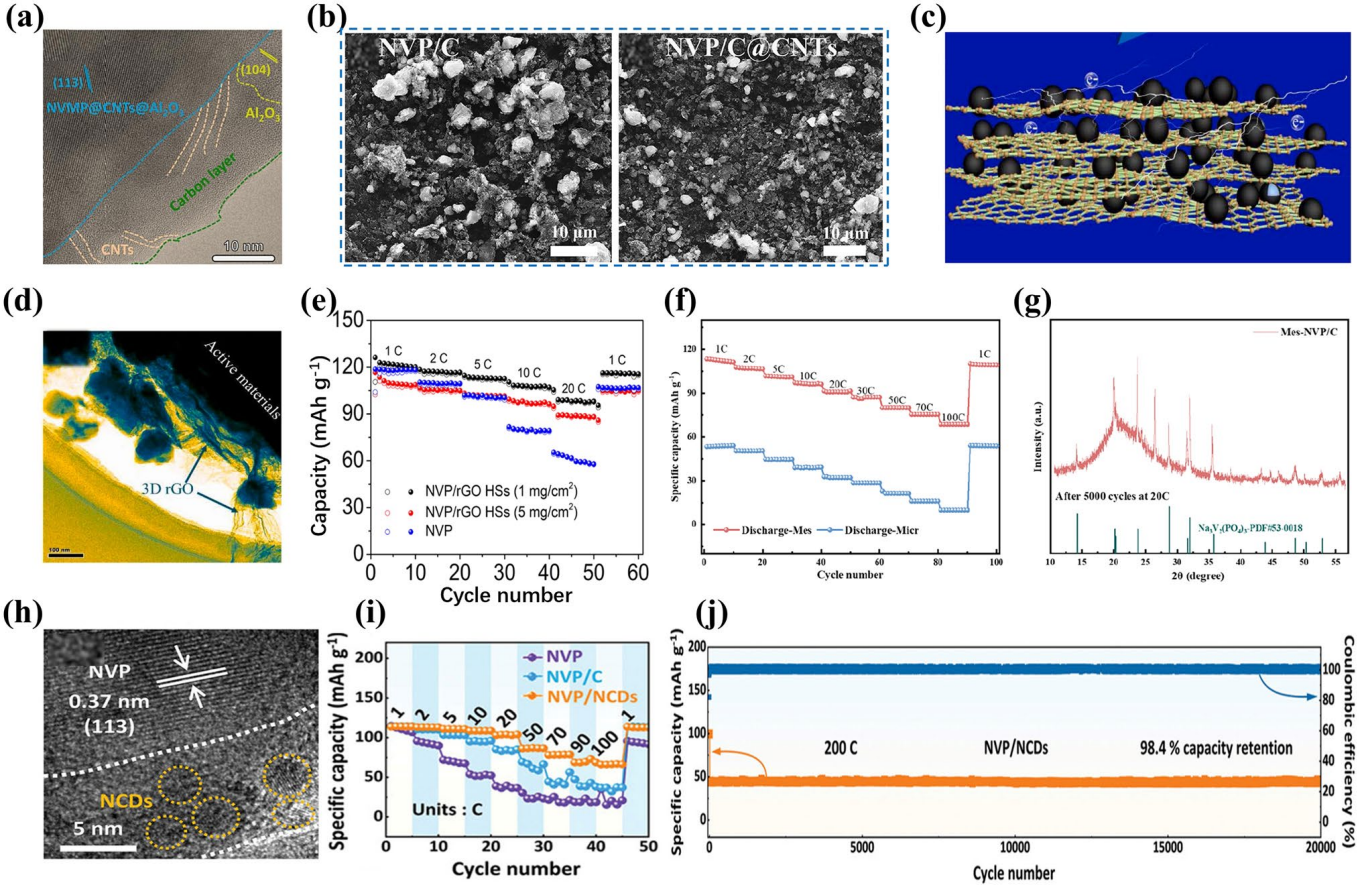
**a** HRTEM image of NVMP@CNTs@1wt%Al_2_O_3_. Reproduced with permission. [99] Copyright 2024, Elsevier. **b** SEM images of NVP/C and NVP/C@CNTs electrodes cycled at 1 C for 100 cycles. Reproduced with permission. [54] Copyright 2021, the Author(s). **c** Schematic illustration and **d** HRTEM image of Nb_0.15_-NVP/C@rGO. Reproduced with permission. [102] Copyright 2022, Elsevier. **e** Rate capability of NVP/G HSs and NVP. Reproduced with permission. [75] Copyright 2020, Elsevier. **f** Rate performance of Mes-NVP/C, **g** XRD (X-ray diffraction) pattern of Mes-NVP/C cycled over 5000 cycles at 20 C. Reproduced with permission. [65] Copyright 2024, American chemical society. **h** HRTEM image of NVP/NCDs, **i** rate performance of NVP/NCDs, **j** cycling performance of NVP/NCDs at 200 C. Reproduced with permission. [96] Copyright 2024, Wiley–VCH

CNTs as a kind of one-dimensional (1D) nanomaterials not only can be characterized with efficient conductive network through entanglement and adhesion but also can avoid accumulation of active grains, which are beneficial to improving the crystalline and electrochemical properties of electrode materials [98]. As depicted in Fig. 4a [99], the CNTs as a bridge between NVP and carbon layer provided highly conductive network for high-speed charge transfer to enhance the electrical conductivity of NVP particles. Dong et al. [54] induced the CNTs to NVP system for preparing NVP/C@CNTs composites by sol–gel method, delivering a high reversible capacity of 97.7 mAh g^−1^ at 0.1 C in contrast with the only 68.7 mAh g^−1^ discharge capacity for NVP/C. As revealed in the SEM images (Fig. 4b), a distinct difference in the NVP/C and NVP/C@CNTs cathodes tested at 1 C after 100 cycles was distinguished, and the distribution of NVP grains kept more homogeneous in the NVP/C@CNTs composite than in the NVP/C.

rGO with unique lamellar morphology, flexibility and high conductivity is commonly used as an ideal two-dimensional (2D) carbonaceous material introduced into electrode materials due to its constructive impacts on mechanical and electrochemical properties [94, 100, 101]. Chen et al. [102] reported that the rGO could form an efficient conductive network to accelerate the electrons migrate in the Ni-doped NVP material (Fig. 4c). Meantime, it also played a tentacle role at the edge of NVP nanoparticles to widen the specific surface area of whole system (Fig. 4d). Xu et al. [75] constructed porous NVP/rGO hollow microspheres by spray-drying approach. The rGO layer effectively elevated the electrons transfer and the unique hollow structure facilitated Na ions accessibility of electrolyte, resulting in a favorable discharge capacity of 116 mAh g^−1^ at 1 C and remarkable rate capacity of 107.5 mAh g^−1^ at 10 C (Fig. 4e).

MC, acted as carrier and support, can provide continuous channels and large surface area with interconnected porous structure, which ensure the fast transport of Na ions and electrons and sufficient electrolyte penetration and accommodate the volume variation during the expansion and contraction of electrode material [103, 104]. Nano-encapsulating of active materials combined with MC has been widely investigated to optimize sodium storage performance [95, 105]. Zhang et al. [65] proposed a ligand-confined growth strategy to confine the ultrasmall NVP nanoparticles in different carbon channels including microporous (Micr-NVP/C), mesoporous (Mes-NVP/C) and macroporous (Macr-NVP/C) carbon channels to seek the optimal carbon channel. The as-prepared Mes-NVP/C with sufficient ion-adsorption sites and well interconnected framework not only provided many convenient pathways for fast diffusion of Na ions and electrons but also effectively suppressed the agglomeration of NVP nanoparticles compared to the Micr-NVP/C and Macr-NVP/C cathode materials. Consequently, the Mes-NVP/C cathode achieved a high specific capacity of 70 mAh g^−1^ at 100 C (Fig. 4f), and exhibited satisfactory cyclic performance (90.48% capacity retention at 20 C after 5000 cycles). The NVP phase of Mes-NVP/C kept a good integrity after 5000 cycles (Fig. 4g), dramatically confirming the desirable ultrahigh-rate cycling stability. It was the nanoconfined NVP materials within mesoporous carbon channels that played a positive role in synchronously boosting rate capability and high-rate cycling stability.

CDs have been ingeniously adopted to integrate with electrode materials in energy conversion and storage fields on account of their fascinating physicochemical properties such as quantum tunneling effect and preeminent conductivity for elevating the electron-transfer ability [106, 107]. Pan’ group [108] adopted a one-step solvothermal approach to synthesize CDs-modified NASICON-structured polyanion cathode material, where CDs were synthesized by hydrothermal method with sucrose as precursor, and revealed that CDs could be viewed as functional additive and nucleation sites to prompt morphology evolution of polyanion cathode material and harvest remarkable rate and cycling performance. Huang et al. [96] reported a facile trace carbon dot incorporation strategy to optimize the structure and sodium storage properties of NVP. The pale-yellow solid CDs were prepared from the mixing solution of tartaric acid, diethylenetriamine and ethanol with the oil bath heating. The as-developed NVP with incorporated CDs (NVP/NCDs) only contained surprisingly low carbon content of 0.76 wt%, and the trace CDs regulated the growth of NVP nanoparticles and significantly balanced the electrical conductivity and electrochemical activity of NVP. As shown in Fig. 4h, the CDs decorated with sufficient surface function groups would form a thinner and more uniform cathode/electrolyte interface (CEI) film combined with carbon coating on the surface of NVP/NCDs, which effectively boosted the interfacial charge diffusion and promoted rate capability significantly. As a result, the NVP/NCDs cathode delivered an impressive reversible capacity of 113.9 mAh g^−1^ at 1 C (Fig. 4i) and exceptional cyclability up to 20,000 cycles corresponding to the capacity retention of 98.4% (Fig. 4j). In addition, ultrafast charging performance was achieved for N/S co-doped CDs/NVP (NVP/NSC) cathode material based on an interfacial synergistic strategy [109], delivering an admirable capacity of 87.2 mAh g^−1^ at 100 C after 10,000 cycles. The assembled full cell NVP/NSC||HC (hard carbon) exhibited an excellent discharge capacity of 115.3 mAh g^−1^ at 5 C and 92.1% capacity retention after 800 cycles.

Generally, a suitable carbon coating layer can serve as armor to shelter NVP particles, which endows the interface contact between NVP and electrolyte with reduced interface impedance, while carbon coating layer has relatively low electrical conductivity in comparison to carbon matrix compositing because the carbon coating layer may hinder the migration of Na^+^ ions and thus affect its electrochemical kinetics [66]. Conversely, for the carbon matrices in the NVP/C, there exists a low impact on lowering contact resistance between NVP and carbon matrix due to the uneven distribution of carbon matrix and the inhomogeneity of NVP particle sizes [43, 97]. Therefore, a synergistic effect can be achieved by combining carbon coating with carbon matrix to further improve electrical conductivity and boost interfacial kinetics for the NVP cathode material.

### Foreign-Ion Doping or Substitution

The carbon coating or modification cannot effectively improve the intrinsic electronic and ionic conductivities of NVP cathode material. To introduce foreign ions into NVP material, as a mainstream strategy, plays a pivotal role to inherently reinforce the bulk-phase intrinsic electronic conductivity, narrow electron transport path and activate more active sites [92]. Generally, the foreign-ion doping or substitution mainly involves the Na, V, and PO_4_^3−^ sites. To be specific, Na site can be replaced by monovalent alkali metal cations and divalent metal ions. Based on the trivalent vanadium V^3+^, the occupation of V site can be divided into isovalent-ion and aliovalent-ion substituting. The replacement of PO_4_^3−^ site can be performed by introducing monoatomic anion or multivalent anion. Additionally, synergistic effects of multiple dimensional doping combined with different sites can significantly boost the electrochemical performance and kinetic properties of NVP as well [110–113].

#### Na Site Doping

There are two kinds of chemical environments for Na ions occupying Na1 site and Na2 site, corresponding to the occupancy rate of 84.3% and 71.9%, respectively [71]. Therefore, the manipulation of Na site's coordination environment by doping metal ions is a useful strategy to elevate Na ion mobility and boost ion-transportation capability in the NASICON-type phosphate cathodes [114].

**Fig. 5:**
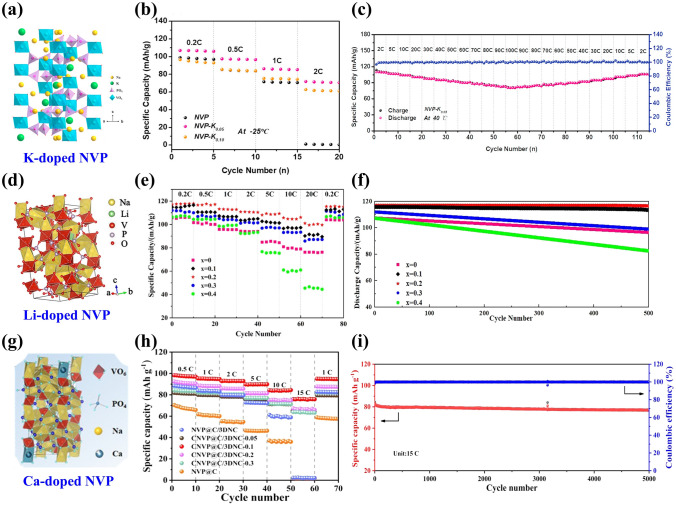
**a** Schematic illustration of crystal structure of K-doped NVP, **b** rate performance of NVP, NVP-K_0.05_ and NVP-K_0.10_ samples at − 25 °C, **c** rate performance of NVP-K_0.05_ at 40 °C. Reproduced with permission [117]. Copyright 2023, Elsevier. **d** Schematic illustration of crystal structure of Na_2.8_Li_0.2_V_2_(PO_4_)_3_, **e** rate performance and **f** cycle performance of Na_3−x_Li_x_V_2_(PO_4_)_3_/C. Reproduced with permission [118]. Copyright 2023, Elsevier. **g** Schematic illustration of crystal structure of Ca-doped NVP, **h** rate capability of NVP@C and CNVP@C/3DNC with different Ca amounts, **i** long-term cycling performance of CNVP@C/3DNC-0.1 at 15 C. Reproduced with permission [121]. Copyright 2022, American chemical society

The K^+^ having a larger radius (1.38 Å) and same number of electronic charges with Na^+^, acted as pillar ion, can broaden Na ion-transport channels and enlarge the unit cell volume through replacing immobile Na ions, presenting a significant enhancement in rate capability and cycling stability of NASICON-type cathodes [115, 116]. The mechanism of K doping in NVP/C was elucidated through theoretical calculations by Yan’s team [67], confirming that the K^+^ activated the activity of Na^+^ by preferentially occupying Na2 site and then Na1 site and optimized the electronic structure by reducing the bandgap of Na_2.9_K_0.1_V_2_(PO_4_)_3_. Moreover, the introduction of K^+^ could expand ion-transport channels, facilitate Na ion's transfer and weaken structural deformation during the fast deintercalation/intercalation processes of Na^+^, harvesting satisfactory sodium storage performance. Shen et al. [117] reported that the K^+^ was employed to dope Na site in NVP framework (Fig. 5a), which promoted Na ion migration, effectively alleviated volume variation and improved structural integrity. Consequently, the as-developed Na_2.95_K_0.05_V_2_(PO_4_)_3_ cathode exhibited splendid sodium storage performance under all-climate (− 25–40 °C) associated with the desirable safety, delivering an initial discharge capacity of 97.8 mAh g^−1^ at 100 C in 40 °C and sustaining a capacity of 72 mAh g^−1^ at 2 C in − 25 °C (Fig. 5b, c). Subsequently, Yan’s team [118] explored the effect of Li doping at Na site by synthesizing a series of Na_3-x_Li_x_V_2_(PO_4_)_3_/C (*x* = 0, 0.1, 0.2, 0.3, 0.4) cathode materials. Figure 5d displays the lattice structure of Li-doped NVP. The introduction of Li^+^ (*x* = 0.2) could activate the activity of Na ions at Na2 sites efficiently and increase structural stability by inhibiting phase transition, rendering an awesome reversible capacity of 116.9 mAh g^−1^ at 0.2 C and 99.82% capacity retention after 500 cycles (Fig. 5e, f).

Apart from the monovalent K and Li doping, high-valence Ca plays a favorable role to occupy part of Na site because of their similar electronegativity (Ca^2+^: 1.16, Na^+^: 1.024) and ionic radius (Ca^2+^: 1.0 Å, Na^+^: 1.02 Å), which significantly improves Na ion-diffusion kinetics and sodium storage performance of NVP material [119, 120]. Chen et al. [121] prepared Na_3-2x_Ca_x_V_2_(PO_4_)_3_ with N-doped carbon nanosheet network (CNVP@C/3DNC-x, *x* = 0.05, 0.1, 0.2, 0.3) by a template-assisted sol–gel route, and the crystal structure of Ca-doped NVP is shown in Fig. 5g. The X-ray photoelectron spectroscopy (XPS) spectra showed clear signature of peaks at 347.3 and 350.9 eV corresponding to the Ca 2*p*_2/3_ and Ca 2*p*_1/2_ spin–orbit states. Meanwhile, there were two V 2*p* peaks at 517.0 and 523.7 eV corresponding to the V 2*p*_2/3_ and V 2*p*_1/2_, indicating that the Ca doping did not change the valence of vanadium and successfully occupied Na sites. Due to the beneficial effects from the regulation of Na site by Ca doping, more Na vacancies generated and thus raised intrinsic electronic conductivity and Na^+^ diffusion kinetics. The optimized CNVP@C/3DNC-0.1 released a superior specific capacity of 83.0 mAh g^−1^ at 15 C with 6.9% capacity decay after 5000 cycles, showing outstanding long-term cycling performance (Fig. 5h, i).

#### V-Site Doping or Substitution

**Fig. 6:**
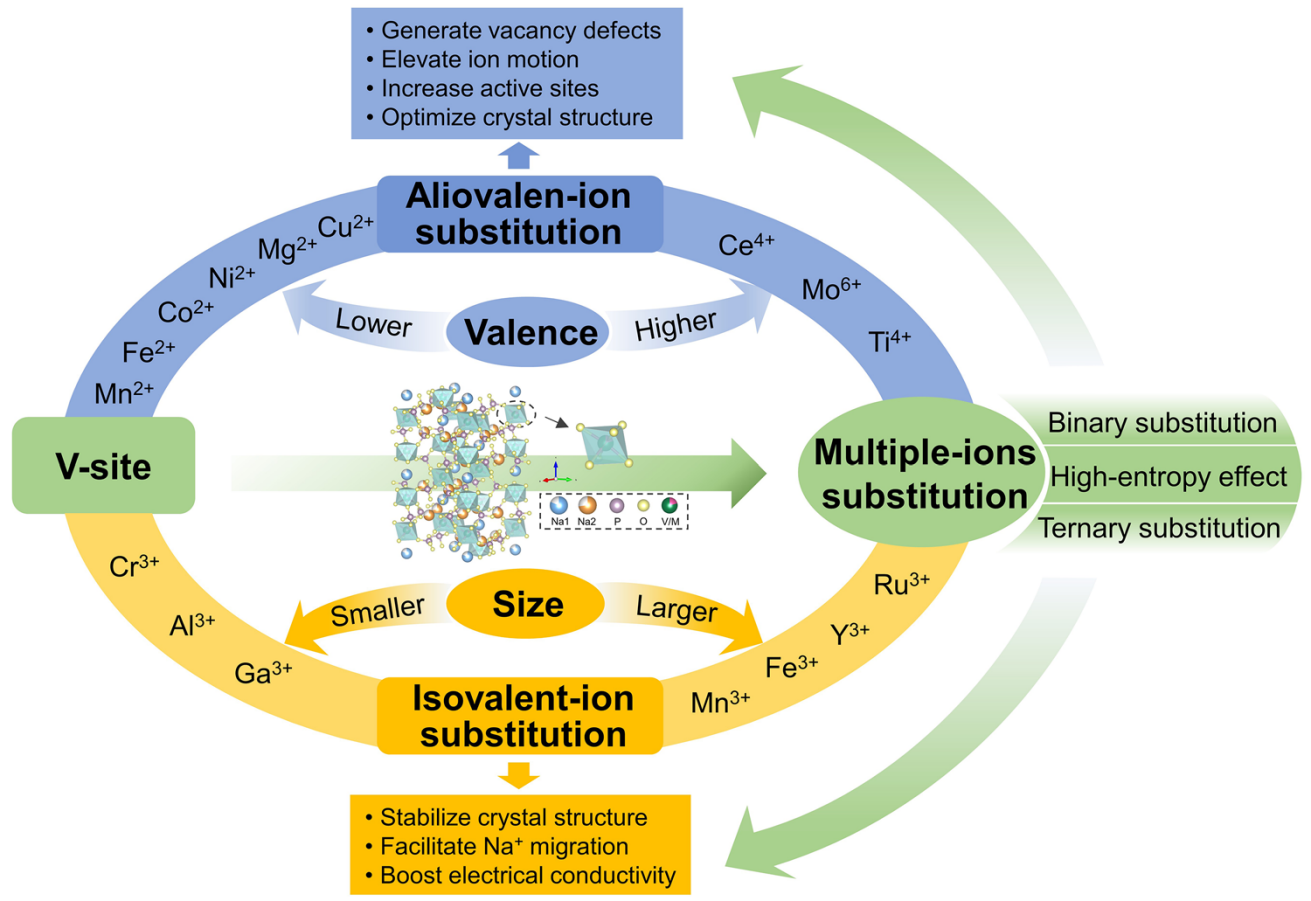
Outline of foreign-ion doping or substitution in V site of NVP material and their advantages

There are mostly various V-substituted NVP cathode materials that have triggered extensive investigations since the substitution of V element holds many merits. Firstly, the replacement of V by inexpensive and nontoxic metal ions can reduce the cost of SIBs [122]. Secondly, it is a desirable thing to achieve satisfactory energy density by virtue of the multivalence states of V element (V^3+^, V^4+^, and V^5+^), which can allow for the reversible multielectron redox reactions at a high-voltage platform [123, 124]. Thirdly, various metal ions gift their own properties which are beneficial to enhancing the stability of structure, increasing reversible discharging capacity and elevating Na^+^ diffusion kinetics of NVP [125–127]. Specifically, the replacement of V in NVP can be divided into three aspects, including isovalent-ion substitution, aliovalent-ion substitution and multiple-ions substitution, as shown in Fig. 6.

##### Isovalent-Ion Substitution

Trivalent metal ion's substitution, presenting electrical neutrality due to the equivalent valence state with V ion, can be easily achieved to optimize the structure of NVP material with fine-tunning property, which slightly impacts the overall structural integrity of NVP. The influences of isovalent-ion substitution are further discussed based on the different sizes of metal ions. For the smaller-size metal ions (Cr^3+^, Al^3+^, Ga^3+^) substituting V, the stronger and shorter M–O chemical bond not only strengthens the oxygen ligand skeleton (MO_6_) for the enhancement of overall structural stability but also accelerates the Na^+^ migration for faster kinetics, while the larger-size metal ions (Mn^3+^, Fe^3+^, Ru^3+^, Sc^3+^, Y^3+^, etc.), acted as pillar ions, play a significant role in supporting the crystal framework and broadening the channels of Na^+^ diffusion, leading to higher ionic conductivity [73, 128–131].

**Fig. 7:**
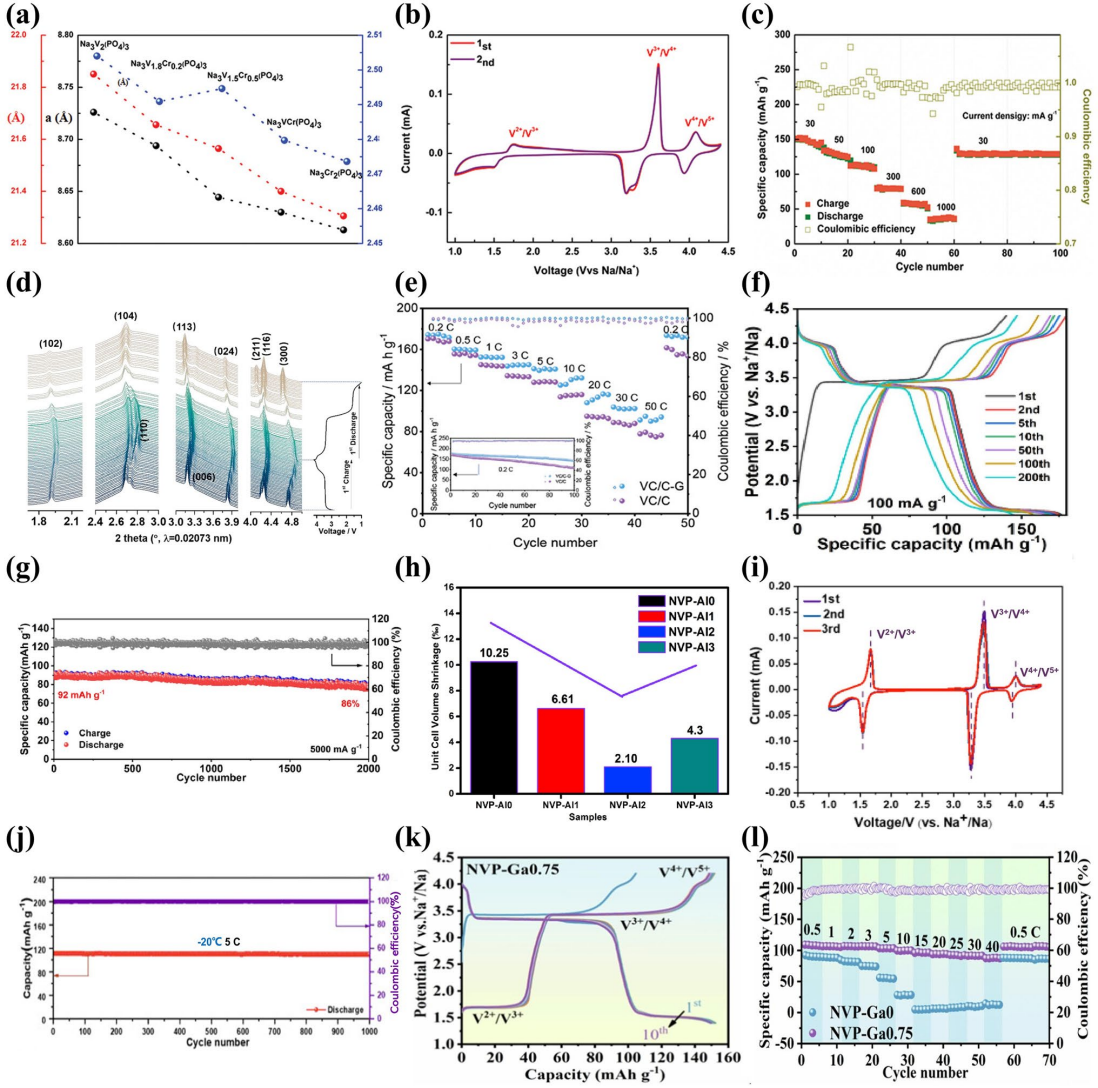
**a** Variations of crystallographic parameters (lattice parameters of *a*, *c* and the ratio of *c*/*a*) for Na_3_V_2−*x*_Cr_*x*_(PO_4_)_3_ (0 ≤ *x* ≤ 2), **b** CV curves of Na_3_V_1.5_Cr_0.5_(PO_4_)_3_ at 0.5 mV s^−1^, **c** rate capability of Na_3_V_1.5_Cr_0.5_(PO_4_)_3_. Reproduced with permission [132]. Copyright 2020, Wiley‐VCH. **d** Operando XRD patterns of Na_3_V_1.5_Cr_0.5_(PO_4_)_3_ material at different charge/discharge states. Reproduced with permission [124]. Copyright 2021, American Chemical Society. **e** Rate properties of VC/C-G and VC/C. Reproduced with permission [134]. Copyright 2022, Wiley‐VCH. **f** Charge/discharge profiles and **g** cycling performance of NVCP@C. Reproduced with permission [136]. Copyright 2023, Wiley‐VCH. **h** Unit cell volume shrinkage of Na_3_V_2-x_Al_x_(PO_4_)_3_/C before and after 50 cycles. Reproduced with permission [47]. Copyright 2017, Elsevier. **i** CV profiles at 0.2 mV s^−1^ and **j** cycling performance of Na_3_V_1.5_Al_0.5_(PO_4_)_3_. Reproduced with permission [142]. Copyright 2022, Elsevier. **k** Charge/discharge curves of NVP-Ga0.75, **l** rate performance within 2.2–4.2 V of NVP-Ga0 and NVP-Ga0.75. Reproduced with permission [61]. Copyright 2023, Elsevier

One of the popular isovalent-ion substitutions in NVP cathode material is Cr^3+^ doping, in which Cr^3+^ possesses a slightly smaller radius of 0.755 Å than V^3+^ (0.78 Å), and it has been corroborated extensively for the great improvement of electrochemical performance [132–135]. Goodenough et al. [132] reported a series of Cr-doped Na_3_V_2−*x*_Cr_*x*_(PO_4_)_3_ (*x* = 0, 0.2, 0.5, 1 and 2) cathode materials. All samples were well consistent with the rhombohedral NASICON structure in space group R $\overline{3 }$ c and no apparent additional diffraction peaks appeared, suggesting a long-range order between the V and Cr in the NVP. Due to the smaller size of Cr, the lattice volume of Cr-doped NVP revealed a decreasing phenomenon (Fig. 7a). It is noteworthy that the Na_3_V_1.5_Cr_0.5_(PO_4_)_3_ exhibited the most stable cycling performance in all samples. Moreover, as shown in Fig. 7b, the Na_3_V_1.5_Cr_0.5_(PO_4_)_3_ cathode enabled three-electron redox reactions with V^2+^/V^3+^ (1.6 V), V^3+^/V^4+^ (3.6 V) and V^4+^/V^5+^ (4.1 V) redox couples fully activated within a wide voltage window of 1.0–4.4 V. Another meaningful observation about Na_3_V_1.5_Cr_0.5_(PO_4_)_3_ was the occurrence of a peculiar solid-solution reaction accompanied by the successful activation of V^4+^/V^5+^, which played a critical role in the stabilization of phase structural evolution during the successive sodiation/de-sodiation processes. Consequently, the Na_3_V_1.5_Cr_0.5_(PO_4_)_3_ manifested a favorable discharge capacity of 150 mAh g^−1^ at 30 mA g^−1^ (Fig. 7c). Furthermore, Chen et al. [124] investigated in depth what a role the Cr behaved in a high redox potential with multielectron reactions and clarified intrinsic reasons for how Cr activated the V^4+^/V^5+^ redox couple. They verified that the Na_3_V_1.5_Cr_0.5_(PO_4_)_3_ involved in a main sodium storage process of solid-solution reaction at high-voltage platforms of 3.4 and 4.1 V through a series of advanced characterization techniques. As displayed in the operando synchrotron-based XRD (Fig. 7d), all of the observed peaks showed well reversible change without asymmetric shifts attributed to the well-maintained polyanionic structure during the Na ion's insertion and extraction processes, indicating that the Cr doping had a positive effect on inhibiting crystal distortion and keeping long-term cyclic stability of NVP cathode material. Besides, with the combination of DFT calculations with electron paramagnetic resonance (EPR) spectroscopy, it was unveiled that the Cr-doped NVP had unpaired electron of Cr in the 3*d* orbital ([Ar]3*d*^5^4*s*^1^) and a narrower forbidden bandgap than undoped NVP (V ([Ar]3*d*^3^4*s*^2^)), which, as the intrinsic reasons, would make contribution to triggering the V^4+^/V^5+^ redox couple and increasing energy density at a high operating voltage. Later, Zhang et al. [134] designed a rGO-supported Na_3_V_1.5_Cr_0.5_(PO_4_)_3_ (VC/C-G) cathode material. Benefiting from the complete activation of multielectron reactions from V^2+^ to V^5+^, the as-designed VC/C-G revealed ultra-stable sodium storage performance, achieving an appreciable energy density of approximately 470 Wh kg^−1^ with a reversible capacity of 176 mAh g^−1^ at 0.2 C (Fig. 7e). Mai’ group [136] reported another Na_3_Cr_4/3_V_3/2_(PO_4_)_3_@C (NVCP@C) cathode material, and they comprehensively clarified that there were two reversible structural evolutions corresponding to solid-solution reaction and two-phase reaction during the charge and discharge processes. It was satisfactory that the NVCP@C exhibited excellent electrochemical performance, yielding an ultrahigh initial discharge capacity of 175 mAh g^−1^ at 100 mA g^−1^ accompanying about 3.1 Na^+^ ions insertion and durable long-term cycling performance with 86% capacity retention at 5000 mA g^−1^ after 2000 cycles (Fig. 7f and g).

Al is an inactive element introduced into NVP material, and it has been demonstrated that Al doping not only particularly stabilizes the phase structure but also impedes the occurrence possibility of side reactions during the charge and discharge processes [137–139]. Masquelier [140] firstly proposed the substitution of V by Al in the Na_3_V_1.5_Al_0.5_(PO_4_)_3_, which realized a comparable increasement of the theoretical energy density (425 Wh kg^−1^) than undoped NVP (396.3 Wh kg^−1^) [141], resulting from the accessibility of higher-voltage V^4+^/V^5+^ redox reaction and lighter weight of aluminum. Chen et al. [47] reported that Al^3+^ partial substitution in V site would provide more volume of interstitial gaps to enable more Na ions migrate due to its smaller ionic radius than V^3+^ (Al^3+^: 0.0535 nm vs. V^3+^: 0.064 nm), as well as significantly strengthen the structural robustness of NVP. It was clear that the cell volume generated distinct contraction after 50 cycles at high discharge rate in different samples. However, the Na_3_V_1.98_Al_0.02_(PO_4_)_3_/C only had a marginal volume change (2.10%), compared with the undoped NVP (10.25%) (Fig. 7h). To improve the operating voltage, Jin’s team [142] investigated the electrochemical reaction mechanism of Na_3_V_1.5_Al_0.5_(PO_4_)_3_ cathode with reversible three-electron redox reactions in the potential range of 1.0–4.4 V. A new redox couple located at around 4.0 V was distinctly witnessed, which was attributed to the initiation of reversible V^4+^/V^5+^ redox reaction (Fig. 7i), resulting in an impressive increase in the capacity (165 mAh g^−1^ at 0.1 C). Moreover, the framework of AlO_6_ octahedron in Na_3_V_1.5_Al_0.5_(PO_4_)_3_ had a more negative average charge of O atom than VO_6_ in Na_3_V_2_(PO_4_)_3_, indicating that the V^3+^ substitution by Al^3+^ made a positive effect on enhancing the oxygen ligand skeleton and elevating the overall structural stability at high potential. As a result, the Na_3_V_1.5_Al_0.5_(PO_4_)_3_ achieved a desirable low-temperature (− 20 °C) cycling performance (98.9% capacity retention after 1000 cycles at 5 C) (Fig. 7j).

Similar to Al element, Ga is another inactive element with ionic radius of 0.062 nm, which can substitute V to obtain improved rate capability and cycling performance [130, 139]. Chen et al. [61] reported that the introduction of Ga^3+^ could successfully activate the V^4+^/V^5+^ redox couple of Na_3_V_2-x_Ga_x_(PO_4_)_3_ (NVP-Gax, *x* = 0, 0.5, 0.75, 1) cathodes within a voltage window of 1.4–4.2 V, containing single-phase and bi-phase electrochemical reactions. A series of spectroscopic and electrochemical tests verified that the modified sample with *x* = 0.75 exhibited a superior structural stability and Ga^3+^ did not participate in the electrochemical reactions. The NVP-Ga0.75 presented a remarkable discharge capacity of 152.3 mAh g^−1^ at 1 C with a favorable energy density of 497.6 Wh kg^−1^ (Fig. 7k). Meanwhile, in 2.2–4.2 V, the NVP-Ga0.75 exhibited excellent rate capability with a high discharge capacity of 105 mAh g^−1^ at 1 C (Fig. 7l).

**Fig. 8:**
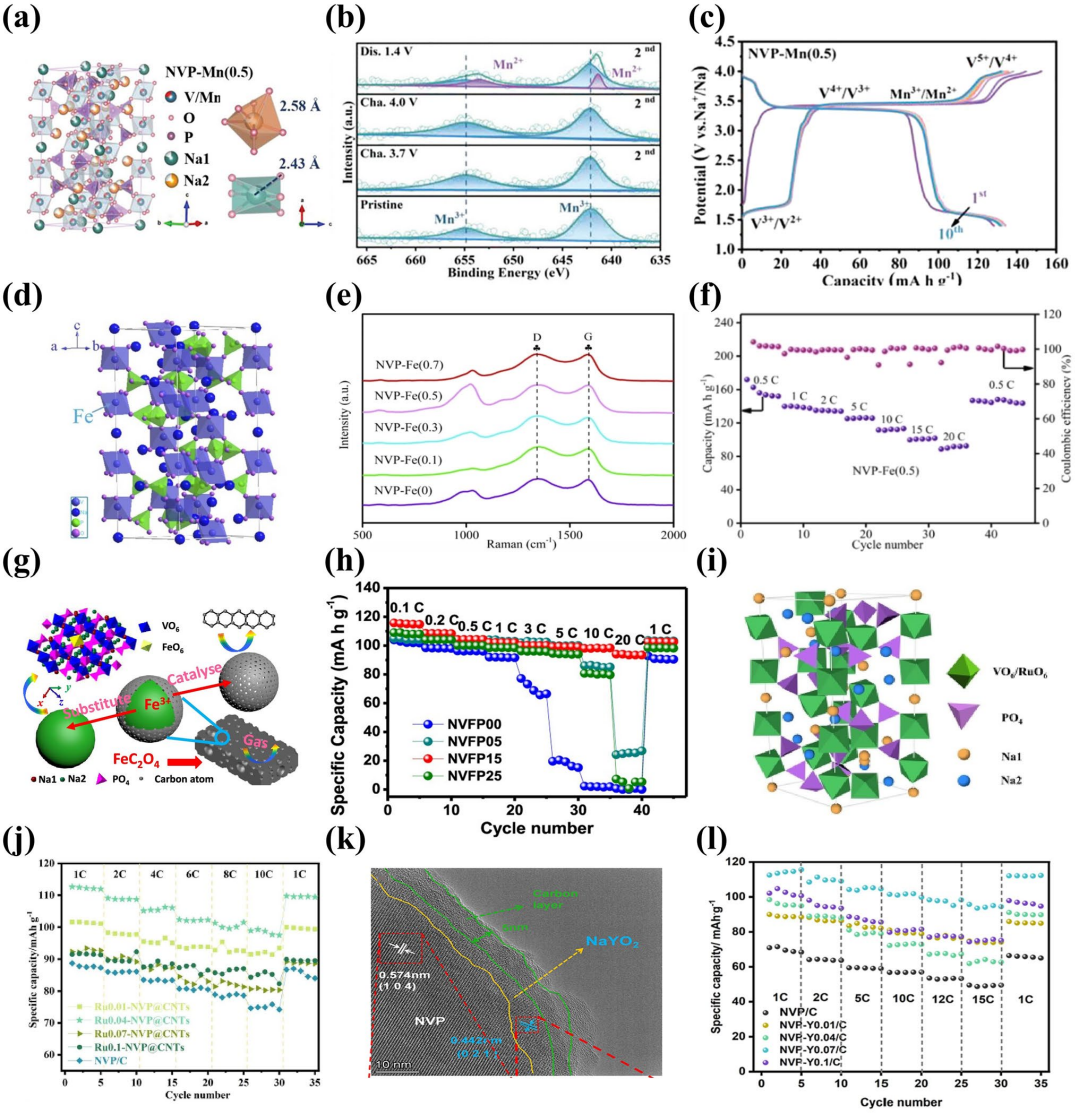
**a** Crystal structure diagram of NVP-Mn(0.5), **b** XPS spectra of Mn 2*p* of NVP-Mn(0.5), **c** charge/discharge curves of NVP-Mn(0.5). Reproduced with permission [129]. Copyright 2023, Wiley‐VCH. **d** Crystal structure diagram of Fe^3+^-doped NVP, **e** Raman spectra of NVP-Fe(x), **f** rate performance of NVP-Fe(0.5). Reproduced with permission [143]. Copyright 2023, Elsevier. **g** Schematic illustration of Fe^3+^ ion into the V sites in NVP, **h** rate capability of different NVFP samples. Reproduced with permission [131]. Copyright 2019, American Chemical Society. **i** Crystal structure model of Ru^3+^ doped NVP, **j** rate capability of different Ru-doped NVP samples. Reproduced with permission [73]. Copyright 2024, Elsevier. **k** HRTEM image of NVP-Y0.07/C material, **l** rate capability of Na_3_V_2-x_Y_x_(PO_4_)_3_/C. Reproduced with permission [145]. Copyright 2023, Elsevier

Among some larger-size metal ions, Fe^3+^ and Mn^3+^ dopants are capable of holding active electrochemical activities, significantly improving the capacity of NVP cathode materials and their doping is beneficial to satisfying the requirement of sustainable development and large-scale applications by virtue of their low-cost and eco-friendly advantages. Additionally, the doping of Fe^3+^ and Mn^3+^ into V site accompanies more than two-electron reactions during the electrochemical reaction process, which availably enhances the energy density of NVP cathodes. Thus, Fe^3+^ and Mn^3+^ doped NVP cathodes have been popularly investigated [129, 143, 144]. Chen et al. [129] performed a family of Mn^3+^ doped Na_3_V_2-x_Mn_x_(PO_4_)_3_ (*x* = 0, 0.1, 0.3, 0.5, 0.7) (NVP-Mn(x)) cathodes by a simple solid-state reaction, and demonstrated that Mn^3+^ could activate multielectron reactions by successive conversions of V^2+^ to V^5+^ in a wide voltage window of 1.4–4.0 V. The crystal structure of NVP-Mn(0.5) is simulated in Fig. 8a. Benefiting from the relatively larger radius of Mn^3+^ (0.785 Å) compared to V^3+^ (0.78 Å), the NVP-Mn(x) cell took lattice expansion, which facilitated Na^+^ migration and enhanced ionic conductivity. Similar to the oxygen ligand surroundings of Na_3_V_1.5_Al_0.5_(PO_4_)_3_, the MnO_6_ of NVP-Mn(0.5) possessed a more negative average charge of O (− 1.51249 e) than the VO_6_ of undoped NVP (− 1.47935 e), significantly strengthening structural stability. In addition, in the hybridized Mn 3*d* orbitals of NVP-Mn(0.5), the electron structure was optimized due to the reduced bandgap and thus improved the electronic conductivity. As illustrated in Fig. 8b, a new redox couple of Mn^2+^/Mn^3+^ was discerned when being discharged to 1.4 V and finally Mn^2+^ disappeared at 3.7 V, indicating Mn^2+^/Mn^3+^ couple participating in the electrochemical reactions. Coupled with the additional charge platforms located around 3.9 V (V^4+^/V^5+^) and 1.7 V (V^2+^/V^3+^) (Fig. 8c), the NVP-Mn(0.5) displayed an appealing reversible capacity of 170.9 mAh g^−1^ at 0.5 C. However, due to the presence of serious Jahn–Teller effect of MnO_6_, it is easy for Mn^3+^ dissolving in electrolyte, posing a dilemma for enhancing cycling stability [140].

For the Fe^3+^ doping, Lin et al. [143] developed Na_3_V_2-x_Fe_x_(PO_4_)_3_/C (*x* = 0, 0.1, 0.3, 0.5, 0.7) ( NVP-Fe(x)) cathode materials via a solid-state reaction. Figure 8d, e revealed the crystal structure and Raman spectra of NVP-Fe(x), indicating that the larger radius of Fe^3+^ had a little impact on the NVP lattice and the NVP-Fe(0.5) carried the highest degree of order and thus would obtain the best electrochemical performance. The NVP-Fe(0.5) cathode material exhibited an exceptional initial capacity of 166.4 mAh g^−1^ at 1 C coinciding with a high energy density of 417.3 Wh kg^−1^ and superior rate performance with 84.6 mAh g^−1^ at 20 C (Fig. 8f), which were attributed to the successful activation of V^4+^/V^5+^ redox couple by Fe^3+^ at a high working voltage to achieve three-electrons reaction. Similarly, Tong’ group [62] synthesized Na_3_Fe_0.8_V_1.2_(PO_4_)_3_/C (NFVP/C) cathode material with an optimal crystal structure by a sol–gel method. With the help of DFT calculations, the NVFP/C showed a suitable ion-migration route with low diffusion barrier. The path 1 (P1) revealed smaller energy barrier of 0.14 eV than the path 2 (P2) (0.30 eV), which remarkably improved Na^+^ diffusion kinetics. Two pairs of redox peaks were observed at 3.4 and 4.0 V, which was in good agreement with two stable platforms in the charge and discharge curves, presenting a high reversible capacity of 115.2 mAh g^−1^ at 0.5 C. Besides, Liu et al. [131] reported that Fe^3+^ could serve as a catalyst to not only effectively modulate carbon layer graphitization and bulk phase for improving electronic conductivity but also accelerate the infiltration of electrolyte for fast diffusion of Na^+^ due to the decomposition of organic iron forming open porous structure (Fig. 8g). Consequently, the optimized Na_3_V_1.85_Fe_0.15_(PO_4_)_3_@C (NVFP) cathode material obtained outstanding rate capability with a discharge capacity of 94.45 mAh g^−1^ at 20 C (Fig. 8h).

Recently, the replacement of other isovalent ions with larger radius than V^3+^ was also explored. Shi et al. [73] investigated the modified mechanism of Ru doping in V site and synthesized a Ru^3+^-doped and CNT-enwrapped Na_3_V_1.96_Ru_0.04_(PO_4_)_3_/C@CNT material (Fig. 8i). On account of the relatively larger ionic radius of Ru^3+^ than V^3+^ (Ru^3+^: 0.68 Å vs V^3+^: 0.64 Å), the modified material broadened and further stabilized the Na ion-migration channels accompanied by higher ionic conductivity and more stable structure with reversibility during the processes of Na^+^ extraction and insertion. The Na_3_V_1.96_Ru_0.04_(PO_4_)_3_/C@CNT had the low value of volume contraction rate that changed less than 3% in the discharge process, further confirming the extraordinary structural stability after the introduction of Ru ion. Consequently, the Na_3_V_1.96_Ru_0.04_(PO_4_)_3_/C@CNT yielded an excellent rate capability with a high capacity of 99.97 mAh g^−1^ at 10 C (Fig. 8j) and superior cycling stability, and submitted an initial capacity of 82.3 mAh g^−1^ and a sustained capacity of 71.3 mAh g^−1^ at 80 C after 14,800 cycles. Subsequently, Shi et al. [88] also proposed that the larger radius Bi^3+^ (1.08 Å) doped into V site would effectively widen ion-transmission channels and promote the Na^+^ diffusion kinetics of NVP. The synthesized Na_3_V_1.97_Bi_0.03_(PO_4_)_3_/C@CNTs cathode revealed superior rate performance, submitting 84.34 mAh g^−1^ capacity at 80 C with 86.96% capacity retention after 6000 cycles. Moreover, Chen et al. [145] firstly reported Y^3+^ doping and successfully obtained Na_3_V_2-x_Y_x_(PO_4_)_3_ (_X_ = 0.01, 0.04, 0.07, 0.1) (NVP-Y_X_) materials. The Y^3+^ possessed a distinctly larger radius of 0.9 Å than V^3+^ (0.64 Å), greatly expanded the Na ion-diffusion pathway and narrowed the crystal vibration, which bolstered Na ion-migration rate and reinforced the crystal structural stability. Notably, a new conductive phase of NaYO_2_, with appealing electric conductivity, emerged between the active material and carbon layer (Fig. 8k), compellingly elevating the ionic conductivity. Consequently, the Na ion-diffusion coefficient of NVP-Y_0.07_ approximately increased one order of magnitude than that of NVP/C. The NVP-Y_0.07_ achieved a high discharge capacity of 125.2 mAh g^−1^ at 0.1 C and sustained 100.4 mAh g^−1^ capacity at 1 C after 300 cycles (Fig. 8l).

##### Aliovalent-Ion Substitution

Although the isovalent-ion doping at V site has a minimal impact on the structural integrity of NVP, the electronic structure of NVP is hardly to further regulation. Thus, the doping of aliovalent ion is popularly appealing, consisting of low-valent ion doping and high-valent ion doping, which have a profound influence on the electrochemical performance of NVP materials. Specifically, the introduction of low-valent ion, thanks to the charge compensation mechanism, will generate favorable hole carriers (or electronic defects) and thus enhance the electronic conductivity, while the high-valent ion will provide additional free electrons and boost electron-transmission ability.

**Fig. 9:**
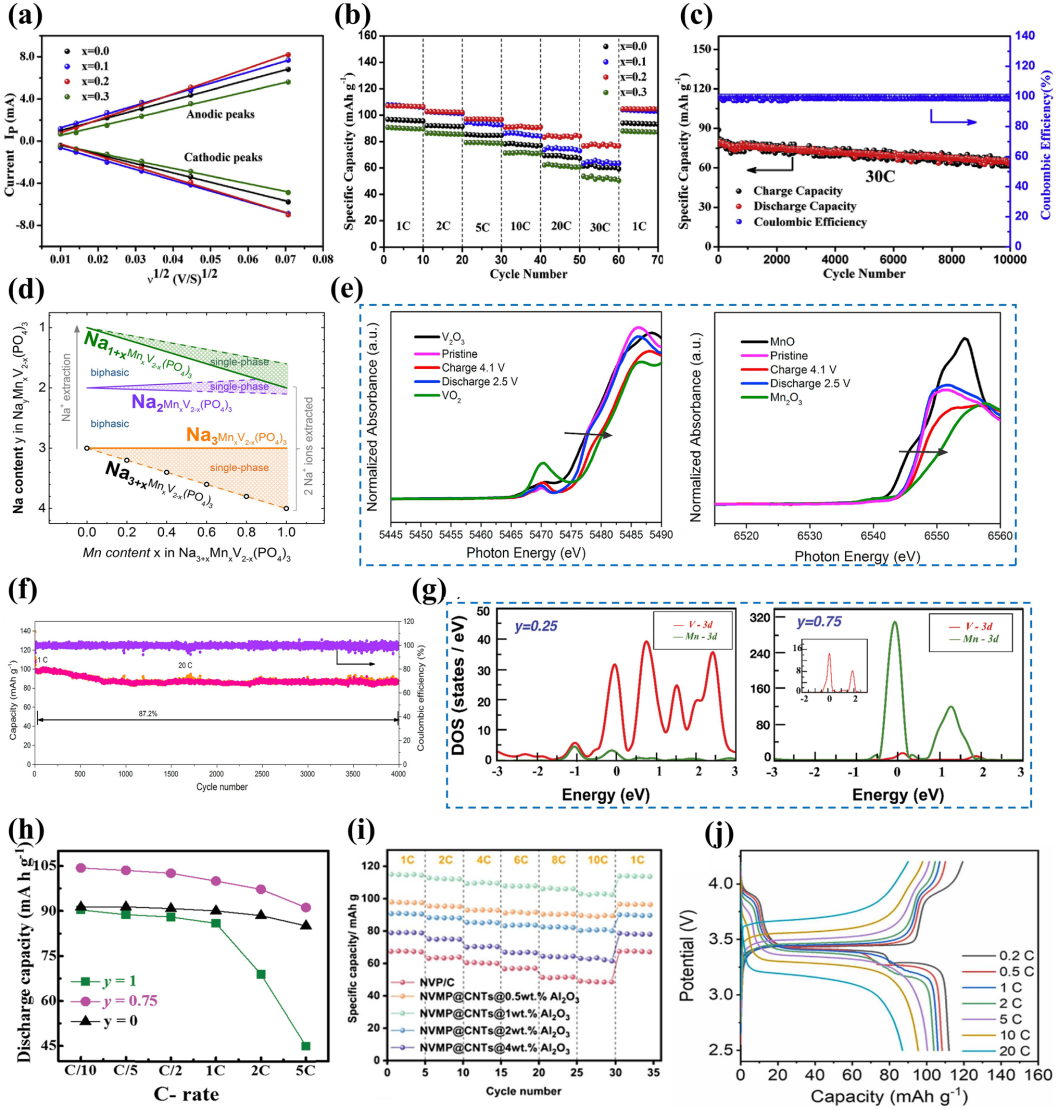
**a** Corresponding relationship between ν^1/2^ and peak current, **b** rate capability of Na_3_V_2-x_Mn_x_(PO_4_)_3_/C cathodes, **c** cycling performance of Na_3_V_1.8_Mn_0.2_(PO_4_)_3_/C at 30 C. Reproduced with permission [149]. Copyright 2018, Elsevier. **d** Schematic illustration of Na^+^ deintercalation regimes from Na_3+x_Mn_x_V_2-x_(PO_4_)_3_ during Na^+^ extraction. Reproduced with permission [150]. Copyright 2020, Elsevier. **e** XANES spectra of (Left)V K-edge and (Right) Mn K-edge of the NVMP/C electrode collected at different charge and discharge states, **f** cyclic stability at 20 C of NVMP/C. Reproduced with permission [152]. Copyright 2018, Elsevier. **g** Projected density of states of Na_3+*y*_V_2−*y*_Mn_*y*_(PO_4_)_3_ cathodes, **h** rate capability of Na_3+*y*_V_2−*y*_Mn_*y*_(PO_4_)_3_ cathodes. Reproduced with permission [151]. Copyright 2019, Wiley‐VCH. **i** Rate capability of different NVMP samples. Reproduced with permission [99]. Copyright 2024, Elsevier. **j** Charge–discharge curves at different rates of NMVP/C@GO. Reproduced with permission [147]. Copyright 2023, Elsevier

Among some divalent metal ions, Mn^2+^ and Fe^2+^, with multielectron transfer potential, have been intensely studied because of their more fruitful properties, and the Mn^2+^ and Fe^2+^ doping not only widen the voltage window but also effectively enhance the sodium storage performance of NVP cathodes [140, 146–148]. The study demonstrated that the existence of Mn^2+^ in Na_3_V_2-x_Mn_x_(PO_4_)_3_ (*x* = 0, 0.1, 0.2, 0.3) materials could stimulate more V^4+^ due to the charge compensation, thereby stabilize the crystal structure and enhance the electronic conductivity. As shown in Fig. 9a–c, the optimal Na_3_V_1.8_Mn_0.2_(PO_4_)_3_/C exhibited the largest Na^+^ diffusion coefficient, superior rate performance with 106.8 mAh g^−1^ at 1 C and impressive cycling stability with 82% capacity retention at 30 C after 10,000 cycles [149]. In addition, Na-rich Na_3+x_Mn_x_V_2-x_(PO_4_)_3_ cathode based on Mn^2+^ substitution and extra Na^+^ manifested great advantages in terms of its desirable electrochemical properties and beneficial phase transformation behavior [150–152]. Zakharkin et al. [150] synthesized a succession of Na_3+x_Mn_x_V_2-x_(PO_4_)_3_ (0 ≤ x ≤ 1) cathode materials, and all Mn-substituted samples exhibited a high-voltage platform at approximately 3.9 V, resulting in that the energy density could be elevated about 8%-10% when x ≥ 0.4 compared with undoped NVP material. As depicted in Fig. 9d, increasing Mn content had a positive impact on the enlargement of a beneficial solid-solution domain linking with sodiated, intermediate and de-sodiated phases. The highly reversible solid-solution phase change in Na_3.5_Mn_0.5_V_1.5_(PO_4_)_3_ (NVMP) was validated [152]. Through the ex situ X-ray absorption near-edge structure spectra (ex situ XANES) and XPS, prominently reversible V^3+^/V^4+^ and Mn^3+^/Mn^4+^ redox couples during the charge and discharge processes were observed (Fig. 9e). More impressively, the NVMP delivered intriguing rate capability with 92.7 mAh g^−1^ at 60 C and exceptional capacity retention of 87.2% after 4000 cycles at 20 C (Fig. 9f). Besides, Ghosh et al. [151] comprehensively investigated the Na-rich Na_3+y_V_2-y_Mn_y_(PO_4_)_3_ (0 ≤ *y* ≤ 1) cathode materials and elucidated the influence of different Mn^2+^ contents on the crystal structure and electrochemical performance. A phase miscibility gap was witnessed at *y* = 0.5, representing two solid-solution regions related to low and high Mn contents. According to the analysis of projected density of states (pDOS) in Fig. 9g, the electronic densities of Mn 3*d* and V 3*d* orbitals in the Na_3.25_V_1.75_Mn_0.25_(PO_4_)_3_ and Na_3.75_V_1.25_Mn_0.75_(PO_4_)_3_ were distinctly different. The Na_3.25_V_1.75_Mn_0.25_(PO_4_)_3_ exhibited a negligible Mn 3*d* electronic density, while the Na_3.75_V_1.25_Mn_0.75_(PO_4_)_3_ was adverse, suggesting that Mn^3+^/Mn^2+^ redox couple was absent and presented in the former and later materials, respectively. Furthermore, Mn element would make more contribution than V element on account of the modulation of Mn redox couples, generating a greater number of valence electrons. The Na_3.75_V_1.25_Mn_0.75_(PO_4_)_3_ cathode revealed high reversible capacities of 100 and 89 mAh g^−1^ at 1 and 5 C, respectively (Fig. 9h). To further ameliorate the poor electronic conductivity of Mn-substituted NVP cathodes, Qian et al. [99] introduced 1 wt% Al_2_O_3_ coating and CNTs in the Na_3_V_5.92/3_Mn_0.04_(PO_4_)_3_ material (NVMP@CNT@1wt%Al_2_O_3_) to curb the dissolution of Mn^2+^ in electrolyte and bolster the electrical conductivity and structural integrity. By the virtue of the effect of Al_2_O_3_ coating and CNTs, the NVMP@CNT@1wt%Al_2_O_3_ cathode performed stable rate capability with 115, 112.8, 110, 107.9, 106.5, 103.0 and 114.09 mAh g^−1^ discharge capacity at 1, 2, 4, 5, 8, 10 and 1 C, respectively (Fig. 9i), and remarkable long-term cycling stability with 84.87% capacity retention after 6000 cycles. Moreover, Li et al. [147] reported a dual carbon-coated Na_3.5_Mn_0.5_V_1.5_(PO_4_)_3_ (NMVP/C@GO) cathode material via a spray-drying route. Thanks to the positive impacts of Mn^2+^ doping and carbon conductive network, the NMVP/C@GO presented a favorable reversible capacity of 112 mAh g^−1^ at 2 C and excellent rate capability with 88 mAh g^−1^ discharge capacity at 20 C (Fig. 9j).

**Fig. 10:**
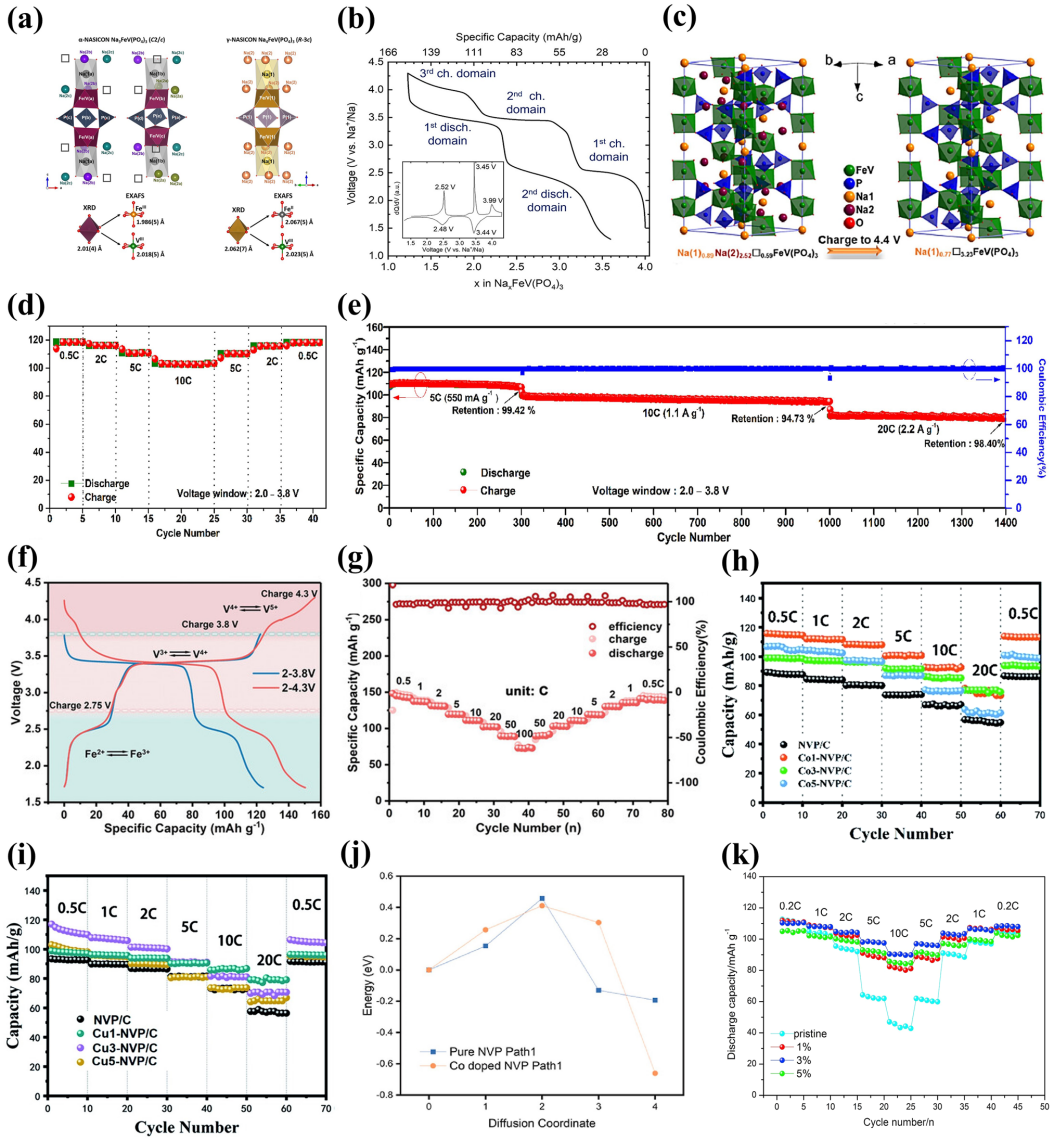
**a** Schematic representations of Na^+^ distributions in Na_3_FeV(PO_4_)_3_ and Na_4_FeV(PO_4_)_3_. Reproduced with permission [156]. Copyright 2021, American Chemical Society. **b** Charge and discharge curves for pre-sodiated Na_4_FeV(PO_4_)_3_ electrode. Reproduced with permission [154]. Copyright 2022, American Chemical Society. **c** Polyhedral representation of NFVP unit cell, **d** rate performance of NFVP electrode, **e** Cycling performance of NFVP at 5, 10 and 20 C. Reproduced with permission [159]. Copyright 2020, Elsevier. **f** Charge–discharge profiles at 0.5 C of NVFP in different voltage windows of 1.7–3.8 and 1.7–4.3 V, **g** rate capability of NVFP in 1.7–4.3 V. Reproduced with permission. [160] Copyright 2023, Wiley‐VCH. Rate performance of **h** Na_3+*x*_V_2−*x*_Co_*x*_(PO_4_)_3_/C, **i** Na_3+*x*_V_2−*x*_Cu_*x*_(PO_4_)_3_/C. Reproduced with permission [161]. Copyright 2021, Royal Society of Chemistry. **j** Energy barriers for NVP and Co-NVP along the diffusion pathway. Reproduced with permission [162]. Copyright 2023, Elsevier. **k** Rate performance of Na_3_V_2-*x*_Ni_*x*_(PO_4_)_3_/C (*x* = 0, 0.01, 0.03 and 0.05). Reproduced with permission [165]. Copyright 2017, Elsevier

For Fe^2+^ doping, Na-rich Na_4_FeV(PO_4_)_3_ cathode material has been widely explored, and the presence of mixed valence of Fe^2+^ and V^3+^ and multielectron redox reactions are contributive to increase meaningful capacity and energy density [153–157]. Masquelier et al. [156] investigated the crystal structure and local chemical environments of Na_4_FeV(PO_4_)_3_ cathode material obtained from the electrochemical sodiation of Na_3_FeV(PO_4_)_3_ material. Through a series of advanced measurements, it was confirmed that Na1 and Na2 sites were almost fully occupied in the Na_4_FeV(PO_4_)_3_ lattice (Fig. 10a). There remained 2.76 Na^+^ per formula unit extracted in the voltage region of 1.3–4.3 V, which was attributed to the successful activation of Fe^2+^/Fe^3+^, V^3+^/V^4+^ and V^4+^/V^5+^ redox couples. The activation of V^4+^/V^5+^ usually had irreversible electrochemical behavior and thus limited the further enhancement of capacity. Subsequently, Masquelier et al. [154] thoroughly investigated the irreversible Na^+^ de/intercalation process in Na_4_FeV(PO_4_)_3_ cathode material through local and bulk perspectives. As displayed in Fig. 10b, during the charge process, there were three potential regions located at around 2.52, 3.45 and 3.99 V relating to the Fe^2+^/Fe^3+^, V^3+^/V^4+^ and V^4+^/^5+^ redox couples, whereas during the discharge process, only two main regions were observed at about 3.44 and 2.48 V, indicating an asymmetric extraction/insertion behavior of Na^+^ at a high potential in the Na_4_FeV(PO_4_)_3_ material. The activation of V^4+^/V^5+^ caused shorter V–O band in a distorted Fe and V local environment, further revealing the asymmetric sodium storage mechanism of Na_4_FeV(PO_4_)_3_ material. Moreover, Xu et al. [158] demonstrated that the reversible activation of V^4+^/V^5+^ mainly relied on the relative contents of Na1 and Na2 in the Fe^2+^-doped NVP lattice, and enough Na2 content effectively ensured the activation of V^4+^/V^5+^. They prepared Na-rich Na_3.4_V_1.6_Fe_0.4_(PO_4_)_3_ cathode material which achieved an extraordinary reversible capacity of 133 mAh g^−1^ in a voltage domain of 2.0–4.1 V, resulting from the successive redox reactions of Fe^2+^/Fe^3+^ and V^3+^/V^4+^/V^5+^. Furthermore, the as-developed Na_3.41_£_0.59_FeV(PO_4_)_3_ (NVFP) sodium-deficient material (Fig. 10c) exhibited an astonishing reversible capacity of 170 mAh g^−1^ at 0.5 C in a wide voltage window of 1.5–4.4 V, showing three pairs of redox peaks located at about 2.55, 3.46 and 4 V coinciding with the Fe^2+^/Fe^3+^, V^3+^/V^4+^ and V^4+^/V^5+^ redox couples, respectively [159]. Together, in a voltage region of 2.0–3.8 V, the NVFP also exhibited excellent rate capability with a discharge capacity of 119 mAh g^−1^ at 0.5 C and extraordinary long cycling life (Fig. 10d, e). It was the admirable electrochemical performance that came from the slight volume change of 2.36% during the successive charging and discharging processes with a solid-solution reaction mechanism. Therefore, increasing output voltage by introducing additional Fe^2+^/Fe^3+^ redox couple and activating high-voltage V^4+/^V^5+^ redox couple could be a significant strategy to harvest satisfactory sodium storage performance. Zhou et al. [160] designed Na_3.5_V_1.5_Fe_0.5_(PO_4_)_3_ (NVFP) cathodes by introducing Fe^2+^ into V site, and the sample emerged two new plateaus at approximately 2.8 and 4.2 V for reversible Fe^2+^/Fe^3+^ and V^4+^/V^5+^ redox couples, respectively (Fig. 10f). The EPR spectra further indicated that the iron with unpaired 3*d* orbital electron made a favorable effect on activating the high-voltage V^4+^/V^5+^ redox couple, as well as the migration paths and electronic structure. As a result, in a voltage window of 1.7–4.3 V, the NVFP cathode showed an awesome discharge capacity of 148.2 mAh g^−1^ at 0.5 C (Fig. 10g), an enviable energy density of 501 Wh kg^−1^ and impressive cycling durability (84% capacity retention after 10,000 cycles at 100 C). Impressively, the assembled full cell HCǀǀNVFP displayed an ultra-stable cycling performance with 63.5% capacity retention after 3000 cycles at 50 C and a material-level energy density of 304 Wh kg^−1^.

Another desirable doping strategy of bivalent transition metal ions is Co^2+^ or Cu^2+^ doping with minor content, and it has been demonstrated that Co^2+^ or Cu^2+^ substitution can sufficiently improve the electrochemical ability of NVP. Chen et al. [161] reported the effects of different contents of Co^2+^ or Cu^2+^ in Na_3+x_V_2-x_M_x_(PO_4_)_3_/C (M = Co^2+^, Cu^2+^; *x* = 0.01–0.05) and fabricated the optimized Na_3.01_V_1.99_Co_0.01_(PO_4_)_3_/C and Na_3.03_V_1.97_Cu_0.03_(PO_4_)_3_/C cathode materials with high discharge capacities of 116 and 114 mAh g^−1^ at 0.5 C, respectively (Fig. 10h, i), which emphasized the low-level amount of dopants playing a crucial role in affecting the electrochemical performance of NVP. Some other Co^2+^-doped NVP cathodes such as Na_3_V_1.93_Co_0.07_(PO_4_)_3_/C were designed via defect regulation by Liu et al. [162], and their study indicated that the Co^2+^ doping not only produced beneficial hole carriers for elevating the electronic conductivity but also enlarged the interplanar spacing to accelerate ionic diffusion rate. Through the theoretical calculations, the introduction of Co^2+^ into V^3+^ site beneficially reduced the bandgap and weakened the energy barrier contributing to fast migration of Na^+^ in NVP (Fig. 10j). Consequently, the Na_3_V_1.93_Co_0.07_(PO_4_)_3_/C composite achieved a high discharge capacity of 114.4 mAh g^−1^ at 0.1 C and a good reversible capacity of 104.6 mAh g^−1^ at 1 C. Besides, Jiang et al. [163] synthesized a succession of Cu-doped NVP/C composites with different contents of Cu dopants (0, 2.5%, 4%, 5% and 6%) via an oxygen vacancies strategy. The incorporation of Cu with oxygen vacancies could generate more electrons and promote the formation of V^4+^ resulting in a shorter V–O bond, which favorably improved the sodium storage performance and kinetic properties of NVP. Furthermore, the optimized cathode with 5% Cu displayed the highest discharge capacity of 111.4 mAh g^−1^ with capacity retention of 90.4% over 300 cycles at 1 C.

Partial substitution of V by Ni element could sufficiently enhance the cell structural stability and ameliorate ion-diffusion dynamics in the NVP structure owing to the introduction of Ni into V site reducing the sizes of VO_6_ octahedrons and PO_4_ tetrahedrons and widening the pathway for Na^+^ diffusion [164]. Zhang et al. [165] prepared Ni-doped NVP/C materials with different content of Ni via a sol–gel method, and the Na_3_V_1.97_Ni_0.03_(PO_4_)_3_/C cathode showed good rate performance with 98.1 mAh g^−1^ at 5 C compared to the pristine NVP/C (64.3 mAh  g^−1^) (Fig. 10k). According to CV results, all Ni-doped samples possessed lower polarization than the pristine sample, and the Na_3_V_1.97_Ni_0.03_(PO_4_)_3_/C with the sharpest and narrowest peaks exhibited enhanced structural reversibility.

**Fig. 11:**
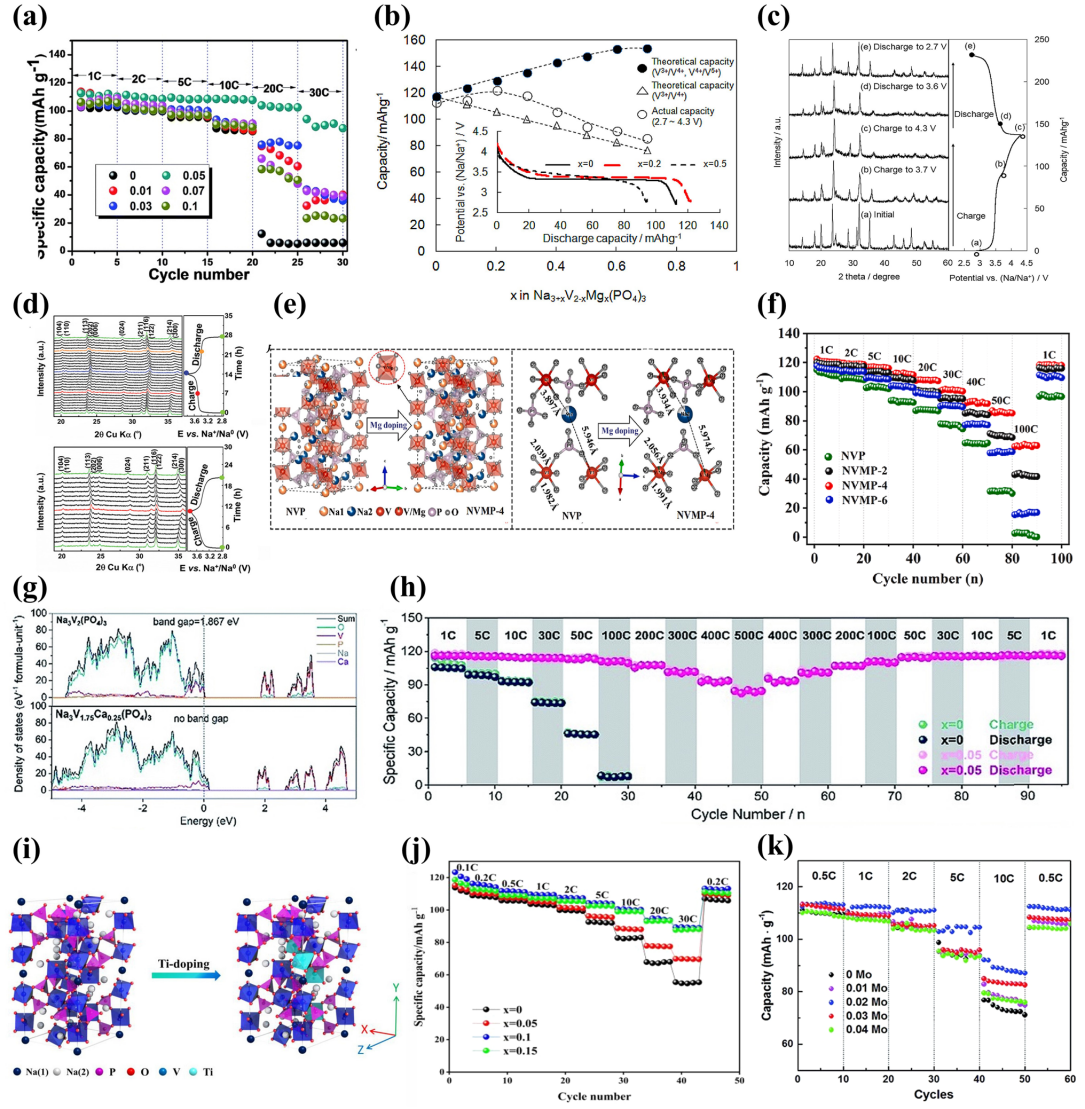
**a** Rate capability of Na_3_V_2−*x*_Mg_*x*_(PO_4_)_3_/C (*x* = 0, 0.01, 0.03, 0.05, 0.07 and 0.1). Reproduced with permission [166]. Copyright 2015, Royal Society of Chemistry. **b** First discharge capacity and theoretical capacity for Na_3+*x*_V_2-*x*_Mg_*x*_(PO_4_)_3_ with different *x* values, **c** XRD patterns of Na_3.5_V_1.5_Mg_0.5_(PO_4_)_3_ at different charge–discharge state. Reproduced with permission [167]. Copyright 2017, Wiley‐VCH. **d** Operando XRD patterns of Na_3+*y*_V_2−*y*_Mg_*y*_(PO_4_)_3_ with *y* = 0.5 (top) and 1 (below). Reproduced with permission [168]. Copyright 2021, Wiley‐VCH. **e** Crystallographic framework of NVP and NVMP-4, **f** rate performance of Na_3+*x*_V_2-*x*_Mg_*x*_(PO_4_)_3_/C. Reproduced with permission [60]. Copyright 2024, Elsevier. **g** Density of states (DOS) for Na_3_V_2_(PO_4_)_3_ and Na_3_V_1.75_Ca_0.25_(PO_4_)_3_, **h** rate capability of Na_3_V_1.95_Ca_0.05_(PO_4_)_3_@C and Na_3_V_2_(PO_4_)_3_@C electrodes. Reproduced with permission [170]. Copyright 2019, Royal Society of Chemistry. **i** Schematic diagram of crystal structure of Ti-doped NVP/C, **j** rate capability of Ti-doped NVP/C. Reproduced with permission [174]. Copyright 2023, American Chemical Society. **k** Rate performance of Na_3−5*x*_V_2−*x*_Mo_*x*_(PO_4_)_3_ (*x* = 0.01–0.04). Reproduced with permission [176]. Copyright 2018, Royal Society of Chemistry

Mg element is a typically inactive element, which is apt to decrease the theoretical capacity of Na_3_V_2-x_Mg_x_(PO_4_)_3_, but additional ionic conductivity and holes produced from Mg^2+^ doping will improve the electrical conductivity. Coupled with Mg^2+^ having a smaller radius of 0.065 nm than V^3+^ (0.074 nm), structural stability is favorably strengthened [166]. The low-dose Mg^2+^-doped Na_3_V_1.95_Mg_0.05_(PO_4_)_3_ material yielded superior rate performance with the specific capacity decreased from 112.5 to 94.2 mAh g^−1^ for increasing rate from 1 to 30 C (Fig. 11 a). To explore the effects of large amounts of Mg dopants, Inoishio et al. [167] investigated a new Na_3+x_V_2-x_Mg_x_(PO_4_)_3_ (*x* = 0.1 to 0.7) family. Interestingly, the Na_3.2_V_1.8_Mg_0.2_(PO_4_)_3_ obtained the highest specific capacity compared to other samples, while the Na_3+x_V_2-x_Mg_x_(PO_4_)_3_ (*x* over 0.3) materials exhibited declined capacities (Fig. 11b). As shown in the ex situ XRD patterns of Na_3.5_V_1.5_Mg_0.5_(PO_4_)_3_ material (Fig. 11c), an irreversible phase transition emerged in the V^4+^/V^5+^ redox region, thereby leading to the lower discharge capacity. Subsequently, Ghosh et al. [168] illuminated the Na^+^ de/intercalation mechanism of Na_3+*y*_V_2-*y*_Mg_*y*_(PO_4_)_3_ (*y* = 0, 0.25, 0.5, 0.75 and 1) cathode materials, and demonstrated that a complete two-phase process would gradually alter to a solid-solution process, along with the Mg^2+^ content increased in the NVP crystal. As performed in the operando XRD studies of Na_3.5_V_1.5_Mg_0.5_(PO_4_)_3_ and Na_4_VMg(PO_4_)_3_ cathodes (Fig. 11d), an intermediate Na_3_V_1.5_Mg_0.5_(PO_4_)_3_ phase emerged, which was accompanied by 0.5 Na^+^ extraction from Na_3.5_V_1.5_Mg_0.5_(PO_4_)_3_ cathode, and the diffraction peaks progressively shifted toward higher 2 $\uptheta$ during the initial charge process, indicating the existence of a solid solution. It was worth noting that the overall cell volume variation only assessed 6.2% compared to the undoped NVP cathode (8.1%), owing to the unextracted Na^+^ increasing the volume of Mg-doped phase and decreasing the lattice mismatch between the Na-rich and Na-poor phases, which significantly boosted structural integrity during cycling process. Conversely, a solid-solution mechanism was observed in the fully sodiated Na_4_VMg(PO_4_)_3_ material over the entire Na de/intercalation range. To further enhance the electrochemical performance of Mg-doped NVP cathode, Zhao et al. [60] prepared Mg^2+^-doped porous flower-like Na_3+*x*_V_2-*x*_Mg_*x*_ (PO_4_)_3_/C (*x* = 0, 0.02, 0.04, 0.06) composites. The optimized Na_3.04_V_1.96_Mg_0.04_ (PO_4_)_3_/C (NVMP-4) lattice model was revealed in Fig. 11e. Based on the ex situ XPS analysis, when being charged to 4.2 V, the Mg^2+^ played a novel role to effectively trigger the partial V^4+^ oxidized to V^5+^ in the NVMP-4 cathode material. Coupled with the hierarchical mesoporous structure with carbon layer, the NVMP-4 exhibited exceptional specific capacity (123.8 mAh g^−1^ at 1 C) and excellent rate performance (73.8 mAh g^−1^ at 100 C) (Fig. 11f).

Additionally, Ca-doped NVP material delivers superior rate capability and cycling performance in comparison to Mg-doped counterpart material [169]. Zhao et al. [170] synthesized Na_3_V_1.95_Ca_0.05_(PO_4_)_3_@C cathode material. The doping of Ca^2+^ with larger radius of 1.00 Å than V^3+^ (0.64 Å) not only enlarged cell volume and lattice spacing to provide broader Na^+^ diffusion channels but also generated mixed valence V^3+^/V^4+^ as charge compensation. The stronger Ca-O bond compared to V–O would remarkably reinforce structural integrity. Through the DFT calculations, the Ca-doped NVP exhibited a disappeared bandgap, lower Na^+^ migration energy barrier and higher lattice energy than the undoped NVP (Fig. 11g), which endowed Na_3_V_1.95_Ca_0.05_(PO_4_)_3_@C cathode with favorable electrochemical performance. The optimized sample delivered unprecedented rate capability with 113 mAh g^−1^ discharge capacity at 50 C and 84 mAh g^−1^ at 500 C, as show in Fig. 11h. Impressively, the Na_3_V_1.95_Ca_0.05_(PO_4_)_3_@C cathode exhibited superior long-term cycling stability with almost 100% capacity retention after 12,000 cycles at 50 C and only 0.0027% capacity decay after 10,000 cycles at 100 and 200 C. Furthermore, Jiang et al. [171] prepared CNTs enwrapped Ca-doped Na_3_V_2-x_Ca_x_(PO_4_)_3_ (*x* = 0.01, 0.04, 0.10, Cax-NVP@CNTs) cathode materials via a facile sol–gel approach. Benefiting from the useful regulation of Ca^2+^ substitution, the Ca0.04-NVP@CNTs had a high initial capacity of 104.3 mAh g^−1^ at 5 C and excellent cycling stability with 99% capacity retention at 50 C after 4000 cycles.

In addition, Ti^4+^ possesses a higher valence and smaller radius than V^3+^, which is beneficial for generating more Na vacancies and enhancing the crystal structure stability. Thus, the Ti^4+^ dopant has been widely employed to substitute V^3+^ for phosphate cathode materials [172, 173]. Ding et al. [174] prepared a series of Na_3_V_2-*x*_Ti_*x*_(PO_4_)_3_/C (*x* = 0, 0.05, 0.1, 0.15) materials through one-step solid-phase method. With increasing the content of Ti^4+^, the crystal volume decreased slightly due to TiO_6_ octahedron being smaller than VO_6_ octahedron, and more vacancies of Na sites were generated for Na^+^ diffusion (Fig. 11i). As a result, when the x = 0.1, the Ti-doped NVP exhibited a satisfactory discharge capability of 123.3 mAh g^−1^ at 0.1 C and excellent rate capability with 89.5 mAh g^−1^ at 30 C (Fig. 11j). Moreover, Huang et al. [175] prepared Na_2.85_V_1.85_Ti_0.15_(PO_4_)_3_ cathode material by a simple solid-phase reaction, delivering a high specific capacity of 101.2 mAh g^−1^ at 10 C with 60% capacity retention after 2000 cycles. Apart from Ti^4+^ substitution, a much higher valence of Mo^6+^ substitution could also improve the sodium storage performance of NVP. Li et al. [176] fabricated a series of Na_3-5*x*_V_2-*x*_Mo_*x*_(PO_4_)_3_/C (*x* = 0, 0.01, 0.02, 0.03, 0.04) cathode materials using a solid-state reaction. Due to the higher valence of Mo^6+^, vacancies would be generated to balance the charge in the NVP system, favorably facilitating the transmission of Na^+^ ion for faster diffusion kinetics. The optimized Na_2.9_V_1.98_Mo_0.02_(PO_4_)_3_/C cathode revealed a superior reversible capacity of 90 mAh g^−1^ at 10 C (Fig. 11k) and better cycling performance with 83.5% capacity retention after 500 cycles.

##### Multiple-Ions Substitution

Although single cationic substitution at V site can effectively improve the electrochemical performance of NVP cathode materials, some limitations still exist. Multiple-ions doping or substitution in NVP can integrate the respective advantages of individual cation and earn favorable electrochemical properties, which covers the binary substitution, ternary substitution and high-entropy effects.

**Fig. 12:**
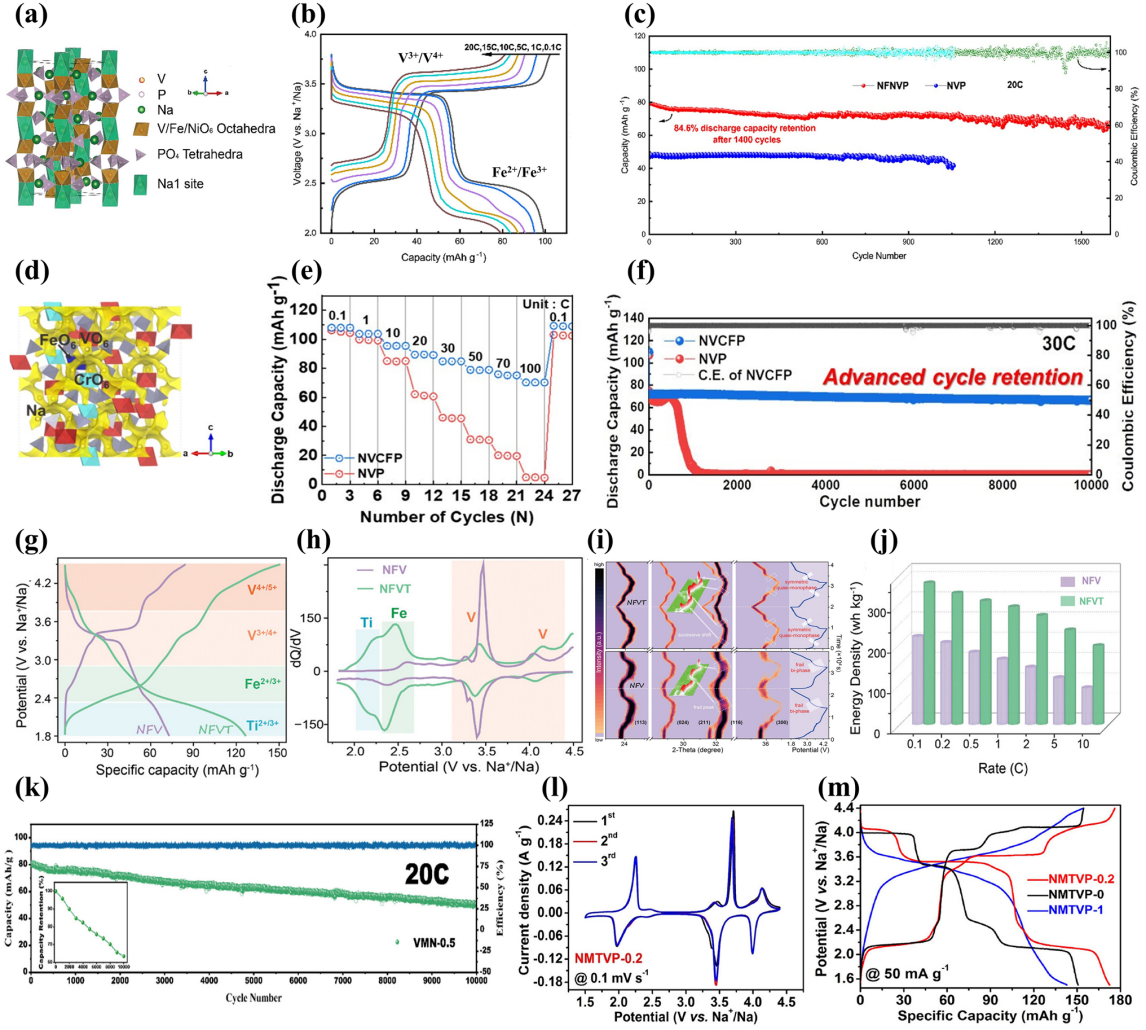
**a** Crystal structure model of NFVNP, **b** charge–discharge curves at different rates of NFVNP in 2.0 − 3.8 V, **c** cycling performance of NFVNP and NVP at 20 C. Reproduced with permission. [140] Copyright 2022, American Chemical Society. **d** Crystal structure model of NVCFP, **e** rate capability and **f** cycling performance of NVCFP and NVP. Reproduced with permission. [110] Copyright 2024, American Chemical Society. **g** Charge–discharge curves at 0.1 C and **h** dQ/dV curves of NFV and NFVT, **i** in situ XRD contour map of NFVT and NFV cathodes along with the first two cycles at 0.1 C, **j** energy density at different rates in NFV and NFVT half cells. Reproduced with permission. [177] Copyright 2024, Wiley‐VCH. **k** Cycle performance of VMN-0.5 at 20 °C. Reproduced with permission. [111] Copyright 2023, Wiley‐VCH. **l** CV curves of NMTVP-0.2, **m** charge–discharge curves of NMTVP-1, NMTVP-0.2 and NMTVP-0.2. Reproduced with permission. [125] Copyright 2023, Wiley‐VCH

For binary substitution, Zhao et al. [140] selected Fe and Ni elements to partially substitute V and synthesized Na_3_Fe_1-*x*_VNi_*x*_(PO_4_)_3_ (*x* = 0, 0.2, 0.4, 0.8) cathode materials by a sol–gel method. The crystal structure of Fe and Ni co-doped NVP (NFVNP) is shown in Fig. 12a. Too much Ni content exerted an adverse impact on the specific capacity. Meanwhile, the Ni doping performed a better improving effect than Fe in declining charge transfer resistance. The modified Na_3_Fe_0.8_VNi_0.2_(PO_4_)_3_ cathode obtained a reversible capacity of 102.2 mAh g^−1^ (close to its theoretical value 105 mAh g^−1^) at 0.1 C in the voltage region of 2.0–3.8 V with two distinct plateaus associated with Fe^2+^/Fe^3+^ and V^3+^/V^4+^ redox reactions (Fig. 12b). The Na_3_Fe_0.8_VNi_0.2_(PO_4_)_3_ cathode displayed favorable low-temperature performance with 89.7 mAh g^−1^ capacity at 2 C and − 10 °C and stable cycling performance with 84.6% capacity retention after 1400 cycles (Fig. 12c). Kim et al. [110] proposed that the Fe and Cr co-doping could significantly enhance structural stability, increase electrical conductivity and optimize phase transition behavior of NVP cathode (Fig. 12d). The optimized Na_3_V_1.5_Cr_0.4_Fe_0.1_(PO_4_)_3_ cathode material achieved ultrahigh-rate capability and outstanding cycle life, delivering a about 71 mAh g^−1^ discharge capacity at 100 C with 95% capacity retention after 10,000 cycles (Fig. 12e, f). Moreover, Zhao et al. [177] investigated the Na_4_FeV_1/3_Ti_2/3_(PO_4_)_3_ (NFVT) cathode and uncovered quasi-monophase behavior in the NFVT material, which was related to multiple electron transfer of Ti^2+^/Ti^3+^, Fe^2+^/Fe^3+^ and V^3+^/V^4+^/V^5+^. Figure 12g displays four regions based on the potential of redox reaction during the charge and discharge processes of NFVT. The potential platform of Fe and Ti tended to be more sloping and the potential platform of V in NFVT was leaner than that in Na_4_FeV(PO_4_)_3_ (NFV), leading to increased specific capacity. Meantime, the additional Ti^3+^ in NFVT was beneficial for the enhancement of Fe^2+^/Fe^3+^ and V^4+^/V^5+^ reactivity (Fig. 12h). The quasi-monophase process in NFVT was studied in depth by in situ XRD in Fig. 12i. Especially, there was obvious differentiation at about 32° in NFV and NFVT accompanied by a frail peak and a successive peak, respectively. As a result, the NFVT with symmetric quasi-monophase presented highly reversible phase transition and an integral crystal structure, driving an ultrafast charging ability (only 3.63 min to reach 80% state of charge at 2 C), stable cycling performance with 0.043% capacity degradation per cycle, and an ideal energy density of over 350 Wh g^−1^ (Fig. 12j).

In addition, the Mn^3+^ substitution in NVP is still plagued with the Jahn–Teller distortion, giving rise to unsatisfactory cyclic stability and rate performance. Therefore, introducing other metal ions to inhibit the Jahn–Teller effect of Mn^3+^ is employed to obtain high-performance NVP cathodes [178]. Chen et al. fabricated a sequence of Na_3.25+*x*_V_1.75-*x*_Mn_*x*_Ni_0.25_(PO_4_)_3_ (*x* = 0, 0.25, 0.5, 0.75, 1, VMN-*x*) cathode materials by Ni and Mn co-substitution. The study demonstrated that Mn^2+^ played a vital role to elevate the reversible capacity attributed to the additional Mn^2+^/Mn^3+^ redox reaction. The inert Ni^2+^ ion acted as a stabilizer was favor for suppressing the Jahn–Teller effect of Mn ions and thus reinforced the crystal structural integrity. The VMN-0.5 exhibited the best electrochemical performance, yielding a high discharge capacity of 108.1 mAh g^−1^ at 0.2 C and an initial capacity of 80.1 mAh g^−1^ at 20 C with 63.5% capacity retention after 10,000 cycles (Fig. 12k). Hu et al. [125] designed Na_3.2_MnTi_0.8_V_0.2_(PO_4_)_3_ (NMTVP-0.2) cathode with reversible 3.2-electrons redox reaction, which contributed from the five redox couples of V^5+^/V^4+^ (≈ 4.1 V), Mn^4+^/Mn^3+^ (≈ 4.0 V), Mn^3+^/Mn^2+^(≈ 3.6 V), V^4+^/V^3+^(≈ 3.4 V) and Ti^4+^/Ti^3+^ (≈ 2.1 V) during the electrochemical reaction process (Fig. 12l). As a result, the NMTVP-0.2 yielded an appealing reversible capacity of 172.5 mAh g^−1^ at 50 mA g^−1^ (Fig. 12m) and an admirable energy density of 527.2 Wh kg^−1^.

**Fig. 13:**
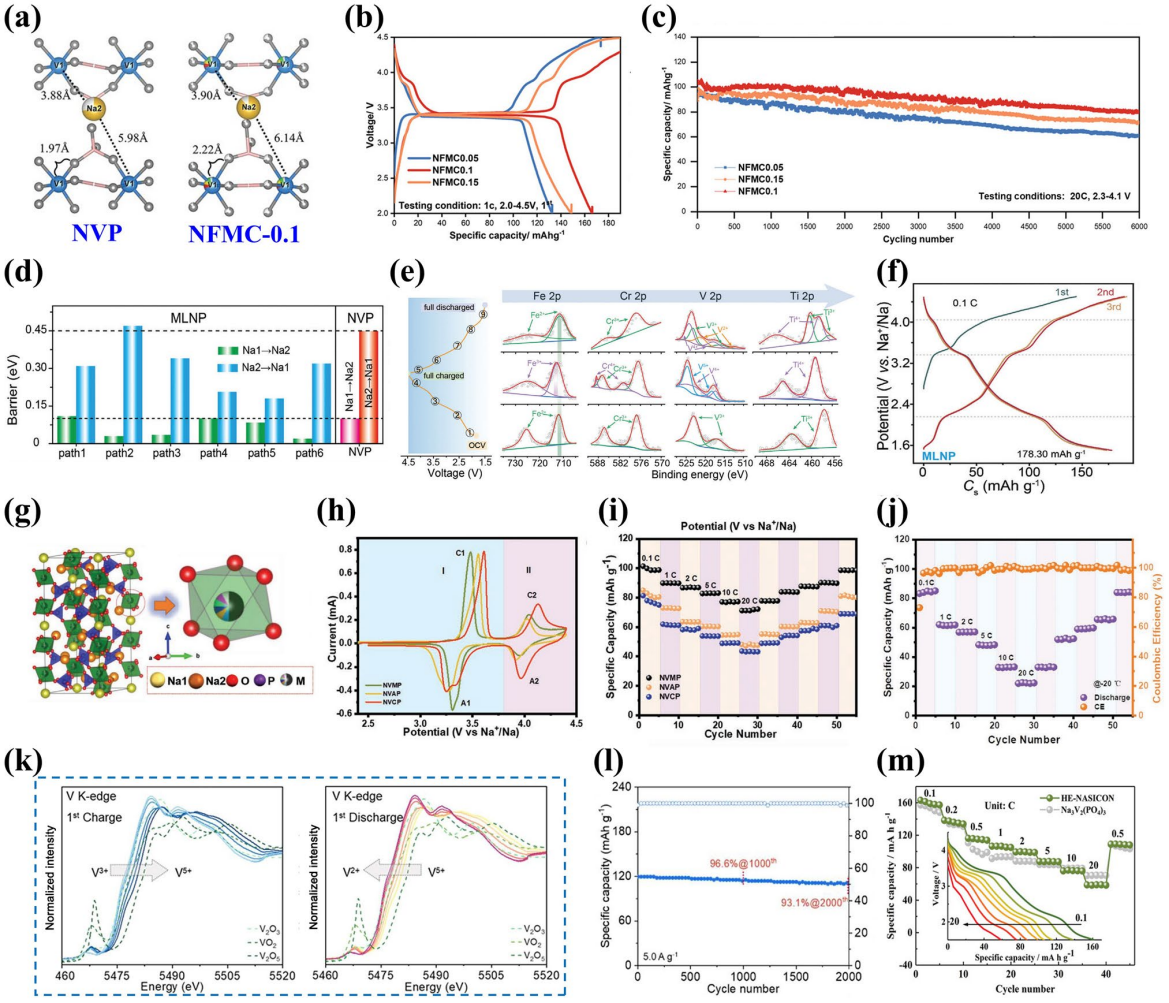
**a** Visualization of crystal structure and atomic occupation of NVP and NFMC0.1, **b** charge–discharge curves and **c** cycling performances of NFMC samples. Reproduced with permission [113]. Copyright 2023, Wiley‐VCH. **d** Comparison of migration energy barriers within different Na^+^ groups of MLNP and NVP, **e** XPS spectra of Fe 2*p*, Cr 2*p*, V 2*p* and Ti 2*p* spectra at different charge/discharge states, **f** charge–discharge curves at 0.1 C of MLNP. Reproduced with permission [126]. Copyright 2024, Wiley‐VCH. **g** Schematic of NVMP crystal structure, **h** CV curves at 0.2 mV s^−1^ and **i** rate performance of NVMP, NVAP and NVCP cathodes, **j** rate capability of NVMP cathode at − 20 °C. Reproduced with permission [180]. Copyright 2023, Wiley‐VCH. **k** V K-edge XANES spectra of HE-V1.6 in charge and discharge processes, **l** cycling performance of HE-V1.6 electrode at 5.0 A g^–1^. Reproduced with permission [181]. Copyright 2024, American Chemical Society. **m** Rate performance of HE-NASICON and Na_3_V_2_(PO_4_)_3_ cathodes. Reproduced with permission [182]. Copyright 2022, Wiley‐VCH

Compared with binary substitution, ternary substitution possesses the ability of multiple dimensional optimized regulation. Sun et al. [113] carried out a fresh type of Fe/Mn/Co co-doped Na_3+2*x*_V_2-3*x*_(FeMnCo)_*x*_(PO_4_)_3_ (*x* = 0.05, 0.1, 0.15, NFMC-x) cathode materials. The heteroatomic introduction of larger ionic radius of Mn^2+^ and Co^2+^ (Mn^2+^: 67 pm, Co^2+^: 74.5 pm, V^3+^: 64 pm) enlarged cell volume and provided plentiful crystal gap to boost Na^+^ diffusion kinetics, and the introduction of smaller radius of Fe^3+^ (55 pm) not only regulated the crystalline state by sustaining the Mn and Co at low state (M^2+^) but also ameliorated lattice distortion originated from the excessive ionic introduction. In the lattice of NFMC-0.1 compared to NVP, as shown in Fig. 13 a, the distance of adjacent Na2 and V increased from 3.88 to 3.90 Å for Na2 and from 5.98 to 6.14 Å for V and the V–O band length in MO_6_ unit increased from 1.97 to 2.22 Å. Therefore, it was the change of M–O band length that was contributive to provide larger removable space for Na ion's migration and gain or loss electrons and perfect the electrochemical performance of NVP. Moreover, the synergy of ternary substitution in NFMC cathodes produced an extra high-voltage platform of 3.8 V associated with the multiple redox couples of Mn^2+^/Mn^3+^/Mn^4+^, Co^2+^/Co^3+^ and V^4+^/V^5+^, leading to increased capacities. Figure 13b presents that the specific capacities of all doped samples surpassed the theoretical capacity (117.6 mAh g^−1^) of NVP, and the NFMC-0.1 cathode material exhibited the best rate capacity with highly structural stability and excellent cycling performance (Fig. 13c). In addition, Gu et al. [126] reported that a homeostatic solid-solution reaction mechanism could be achieved by diversifying reaction pathways in the as-prepared Na_3.5_Fe_0.5_V_0.5_Cr_0.5_Ti_0.5_(PO_4_)_3_ (MLNP) cathode material and combining the merits of progressive TM ions (V, Fe, Ti and Cr) with multilevel redox couples, which overcame the high barrier of bottleneck of V^4+^/V^5+^ at high voltage. In the reciprocal migration of Na1 ↔ Na2 course, the process of Na1 → Na2 (path1) was sluggish with endothermic process, but the Na2 → Na1 (path2) was exothermic with low barrier, indicating that the absorb of inactive Na1 was more difficult than its release. As shown in Fig. 13d, the MLNP with lower migration energy barriers of pathways than NVP further encouraged the absorb of inactive Na1, enabling a higher capacity. The different migration modes combined with different sites were attributed to the various TMO_6_ octahedra in MLNP accompanying multilevel redox reactions, compared to only VO_6_ in NVP. Through the ex situ XPS analysis in Fig. 13e, a successive oxidization course of Ti^3+^/Ti^4+^, Fe^2+^/Fe^3+^, V^3+^/V^4+^ and Cr^3+^/Cr^4+^ in the charge process and a successional reduction course of Cr^4+^/Cr^3+^, V^5+^/V^4+^, V^4+^/V^3+^, Fe^3+^/Fe^2+^, Ti^4+^/Ti^3+^ and V^3+^/V^2+^ in the discharge process were observed, effectively propelling sufficient Na storage and enhancing reversible capacity in subsequent cycling. The variation of cell volume was barely 1.73% in MLNP material. Moreover, the MLNP cathode delivered a high reversible capacity of 178.30 mAh g^−1^ at 0.1 C (Fig. 13f), met with the pursuit of energy density reaching 440 Wh kg^−1^, and exhibited fascinating cycling durability with 0.0061% capacity decay per cycle for 500 cycles at 5 C.

Recently, to further improve the electrochemical performance of NVP, a high-entropy concept involving the incorporation of five or more TM elements with apt molarity is employed to optimize NASICON-type cathode materials [112]. High-entropy substitution is beneficial for the optimization of crystal structure resulting from the chemical orders for mixed cations and generated vacancies [179–181]. Li et al. [180] designed a high-entropy Na_3_VAl_0.2_Cr_0.2_Fe_0.2_In_0.2_Ga_0.2_(PO_4_)_3_ (NVMP) cathode material by introducing five metal elements (Al, Cr, Fe, In, Ga) into V site (Fig. 13g), aiming to suppress the irreversible phase transition at the voltage range of over 4 V. Two redox pairs corresponding to V^3+^/V^4+^ and V^4+^/V^5+^, presenting lower polarization and higher reversibility of Na^+^ de/intercalation compared to Na_3_VAl(PO_4_)_3_ (NVAP) and Na_3_VCr(PO_4_)_3_ (NVCP), were distinguished in Fig. 13h. According to the ex situ XRD measurement, all characteristic peaks exhibited high reversibility during the charge and discharge processes, disclosing the crystal structural evolution of NVMP corresponding to a beneficial solid-solution behavior for improving electrochemical reversibility and structural robustness. The high-entropy effect played a pivotal role to inhibit the irreversible phase transition caused by high potential V^4+^/V^5+^ redox reaction. Thereby, the NVMP cathode with a minimal cell volume change of 1.1% achieved a high discharge capacity of 102 mAh g^−1^ at 0.1 C (Fig. 13i) and could obtain satisfactory rate performance (83 mAh g^−1^ reversible capacity 0.1 C) even at an extreme temperature of − 20 °C (Fig. 13j). Hao et al. [181] reported a Na_3.32_V_1.6_Cr_0.08_Fe_0.08_Mn_0.08_Mg_0.08_Ca_0.08_(PO_4_)_3_ (HE-NVP) cathode material via a simple sol–gel approach. In combination of in situ XANES and in situ XRD results, a successful reversible activation of V^4+^/V^5+^ redox couple (Fig. 13k), a solid-solution reaction and a biphasic conversion reaction accompanied by highly structural reversibility were confirmed. The HE-NVP afforded a remarkable discharge capacity of 120 mAh g^−1^ at 5.0 A g^−1^ with 93.1% capacity retention after 2000 cycles (Fig. 13l). Moreover, some other high-entropy polyanionic cathode materials such as Na_3.4_Fe_0.4_Mn_0.4_V_0.4_Cr_0.4_Ti_0.4_(PO_4_)_3_ (HE-NASICON) were proposed by Li et al. [182]. Their research unveiled that the HE-NASICON achieved multi-cationic redox reactions and mitigated the structural degradation in a wide voltage window (1.5–4.5 V), enabling a superior reversible capacity of 161.3 mAh g^−1^ at 0.1 C (Fig. 13m), along with a robust structural stability.

#### ***PO***_***4***_^***3−***^***-Site Doping or Substitution***

Compared to the replacement of Na and V sites, research on the substitution of inactive anion PO_4_^3−^ in NVP is less. PO_4_^3−^-site doping or substitution, as a feasible route, can also effectively retard the capacity loss and enhance cycling stability. At present, anion doping is mainly concluded to two aspects, the monoatomic anion F^−^ substitution and the multivalent anion substitution such as SiO_4_^4−^ [183], SO_4_^2−^ [184], BO_3_^3−^ [185, 186] and MoO_4_^2−^ [187]. Generally, the F^−^ substitution has been widely explored compared with multivalent anion substitution [53, 185].

##### Monoatomic Anion Substitution

**Fig. 14:**
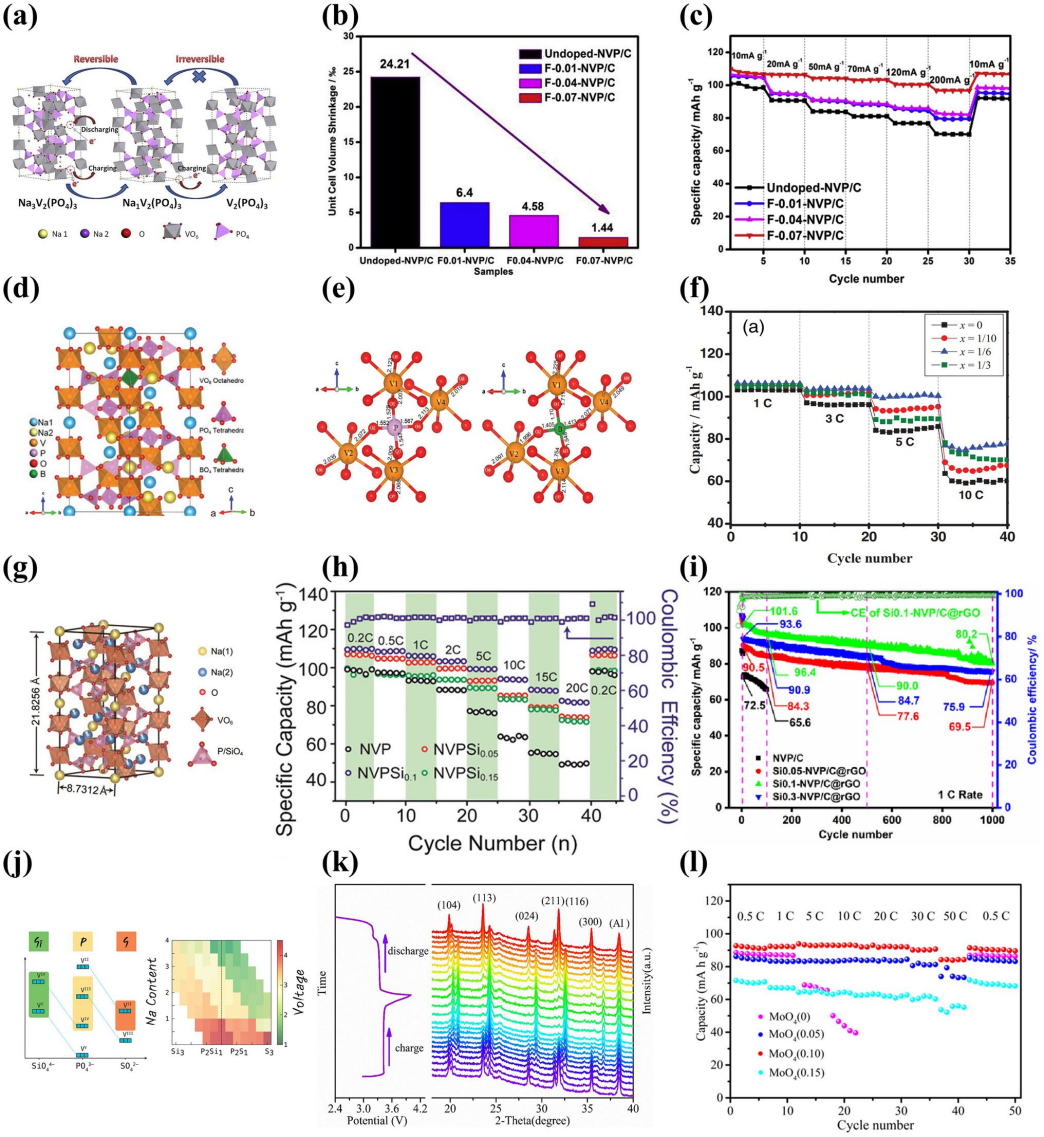
**a** Schematic diagram of structural degradation for Na_3_V_2_(PO_4_)_3_, **b** percentage of unit cell volume shrinkages before and after 1000 cycles at 200 mA g^−1^ and **c** rate capability of Na_3_V_2_(PO_4_)_3-x_F_x_/C (*x* = 0, 0.01, 0.04 and 0.07) composites. Reproduced with permission [191]. Copyright 2017, Wiley‐VCH. **d** Crystal structure of Na_3_V_2_P_3–*x*_B_*X*_O_12_ (*x* = 1/6), **e** local distortion around the doping site of Na_3_V_2_P_3*x*_B_*x*_O_12_ (Left) (*x* = 0) and (Right) (*x* = 1/6), **f** rate capability of Na_3_V_2_P_3-*x*_B_*x*_O_12_ (*x* = 0, 1/10, 1/6, 1/3) electrodes. Reproduced with permission [185]. Copyright 2017, Wiley‐VCH. **g** Schematic illustration of crystal structure NVPSi_0.1_, **h** rate performance of NVPSi_*x*_ (*x* = 0, 0.05, 0.10, 0.15). Reproduced with permission [183]. Copyright 2020, Wiley‐VCH. **i** Cycling performance of Na_3+*x*_V_2_(PO_4_)_3-*x*_(SiO_4_)_*x*_/C@rGO (*x* = 0.05, 0.1, 0.3) at 1 C. Reproduced with permission [195]. Copyright 2020, Wiley‐VCH. **j** Multicomponent Na-V-P-(Si/S)-O quinary phase diagram. Reproduced with permission [184]. Copyright 2022, American Chemical Society. **k** XRD patterns of NVP-MoO_4_ (0.10) electrodes during the first charge–discharge cycle, **l** rate capability of NVP-MoO_4_ (*x*) cathodes. Reproduced with permission [187]. Copyright 2021, Elsevier

Due to the anion F^−^ featured by strong electronegative and inductive effect, a higher redox potential could be obtained during the charge and discharge processes for the F-doped NVP cathodes, which considerably boosted sodium storage performance [188–190]. Chen et al. [191] synthesized a series of F-doped Na_3_V_2_(PO_4_)_3-*x*_F_*x*_/C (*x* = 0, 0.01, 0.04, 0.07) cathode materials via a simple solid-state method. They demonstrated that the F-substitution not only mitigated the structural degradation from irreversible Na_3_V_2_(PO_4_)_3_ to V_2_(PO_4_)_3_ (Fig. 14 a), but also effectively shortened the pathways of Na^+^ ion and electron transportation. The variation of cell volume of F-doped NVP cycled at 200 mA g^−1^ for 1000 cycles is calculated in Fig. 14b, exhibiting strikingly declined shrinkage of cell volume in all F-doped NVP materials compared to NVP. Therefore, the optimized Na_3_V_2_(PO_4_)_1.93_F_0.07_ cathode material achieved enhanced structural stability and improved Na^+^ kinetics behavior, rendering better rate capability with a good discharge capacity of 113 mAh g^−1^ at 10 mA g^−1^ (Fig. 14c) and 86% capacity retention after 1000 cycles at 200 mA g^−1^. Subsequently, Chen et al. [192] reported a F-doped and V-defects Na_3_V_1.98_(PO_4_)_1.97_F_0.3_/C cathode with an extra potential platform at about 4.0 V corresponding to the V^4+^/V^5+^ redox pair, presenting 116.9 mAh g^−1^ reversible capacity at 0.1 C and an impressive D_Na_^+^ of 3.66 × 10^–13^ cm^2^ s^−1^ which was three orders of magnitude higher than that of undoped NVP/C material (7.41 × 10^−16^ cm^2^ s^−1^).

##### Multivalent Anion Substitution

It is the multivalent anion substitution that is supposed to be more effective and feasible to realize the precisely optimized preparation owing to the more similar anionic structure in comparison to the monoatomic anion F^−^ [183]. Except for the BO_3_^3−^ holding triangle ionic structure, the ionic structures of SiO_4_^4−^, SO_4_^2−^ and MoO_4_^2−^ are identical with the tetrahedral PO_4_^3−^, presenting better electrochemical performance by virtue of the structural compatibility than BO_3_^3−^ [185, 187, 193, 194]. Hu et al. [185] comprehensively investigated the evolution of crystal and electronic structure of Na_3_V_2_(PO_4_)_3-x_(BO_3_^3−^)_x_ (0 ≤ x ≤ 1) cathode materials. It was unveiled that the BO_3_^3−^ substitution played a critical role to optimize crystal structure and effectively narrow the bandgap in a new energy state. As manifested in Fig. 14d, the BO_3_^3−^ would occupy the tetrahedral interstitial site, which almost altered the framework of the optimized Na_3_V_2_(PO_4_)_3–1/6_(BO_3_)_1/6_ material. Additionally, the strength of B-O bond was much weaker than that of P-O bond and the V–O band length was marginally reduced owing to the substitution of B (Fig. 14e), promoting the migration of Na^+^ ions. The Na_3_V_2_(PO_4_)_3–1/6_(BO_3_)_1/6_ exhibited improved discharge capacities of 100 and 70 mAh g^−1^ at 5 C and 10 C, respectively (Fig. 14f). Qiu et al. [186] also pointed out the profound influence of BO_3_^3−^ substitution on the phase transition and electrochemical reactions of NVP. The anion group BO_3_^3−^ into PO_4_^3−^ site played three roles to expand the domain of solid solution, accelerate the structural transformation to V^2+^-containing phase and mitigate the short-scale heterogeneity of P and Na nucleus.

Introducing isostructural SiO_4_^4−^ into NVP framework would greatly optimize NVP lattice and improve its rate capability and cyclic stability. To be specific, the larger radius and lower electronegativity of Si^4+^ compared to P^5+^ (Si^4+^: 0.024 nm vs. P^5+^: 0.017 nm; electronegativity: Si^4+^: 1.90 vs. P^5+^: 2.19) rendered more stronger Si–O bond than P-O bond, which enhanced structural stability. Furthermore, the produced structural variation would broaden the ion's diffusion pathways and thus increase electrical conductivity. The as-synthesized Na_3.1_V_2_(PO_4_)_2.9_(SiO_4_)_0.1_ cathode (Fig. 14g) impressively obtained an increased occupancy ratio at Na2 site from 0.7090 to 0.7395, which harvested higher Na storage capacity with a higher specific capacity of 82.5 mAh g^−1^ at 20 C compared to undoped NVP with only 49.7 mAh g^−1^ (Fig. 14h) [183]. Chen et al. [195] synthesized Na_3.1_V_2_(PO_4_)_2.9_(SiO_4_)_0.1_/C@rGO cathode material, and demonstrated that the SiO_4_^2−^ not only widened the transportation channels of Na^+^ but also was beneficial for the formation of vesicular structure, which enlarged contact region between active material and electrolyte. Coupled with the advantages of rGO, the optimized cathode delivered a favorable discharge capacity of 113.6 mAh g^−1^ at 0.1 C and good cyclic stability with 79.0% capacity retention after 1000 cycles at 1 C (Fig. 14i). In combination of DFT calculations and thermodynamic analysis, Kapoor et al. [184] exploited the different effects of the partial or full substitution of PO_4_^3−^ by SiO_4_^4−^ or SO_4_^2−^ and unlocked the uncharted multicomponent Na-V-P-(Si/S)-O quinary phase diagram (Fig. 14j). Specifically, the SO_4_^2^-doping could raise the voltage of each V redox pairs including V^2+^/V^3+^, V^3+^/V^4+^ and V^4+^/V^5+^ at the cost of the massive amount of Na^+^ intercalated. But owing to the enlargement of low voltage of V^2+^/V^3+^, the overall average voltage would be lower. Oppositely, the replacement of PO_4_^3−^ by a dose of SiO_4_^4−^ dopants would activate the high voltage of V^4+^/V^5+^ and thus enhance the average voltage without necessary extraction of Na from the Na1 site, which was not experimentally accessible for the activation of V^4+^/V^5+^ in practical NVP composites.

Furthermore, Liu et al. [187] firstly proposed that MoO_4_^2−^ partially replacing PO_4_^3−^ could also propel Na^+^ diffusion kinetics and sodium storage performance. They developed a succession of Na_(3+2*x*)_V_2_(PO_4_)_(3-*x*)_(MoO_4_)_(*x*)_ (NVP-MoO_4_(*x*), *x* = 0, 0.05, 0.10, 0.15) cathode materials via a convenient solid-state reaction. The result of pDOS revealed the effect of MoO_4_^2−^ substitution, and the modified NVP-MoO_4_(0.1) sample obtained a reduced bandgap (3.67 eV) compared to NVP-MoO_4_(0) (4.97 eV) accompanied by some newborn energy states at the bottom of conduction band, which further optimized the local lattice structure and electronic structure of NVP. Meantime, the in situ XRD measurement presented highly structural reversibility of NVP-MoO_4_(0.1) sample during the subsequent electrochemical cycling process (Fig. 14k). Consequently, the obtained NVP-MoO_4_(0.1) cathode performed a dramatic improvement in the rate capability with 84.2 mAh g^−1^ discharge capacity at 50 C (Fig. 14l).

#### Multi-Sites Doping or Substitution

**Fig. 15:**
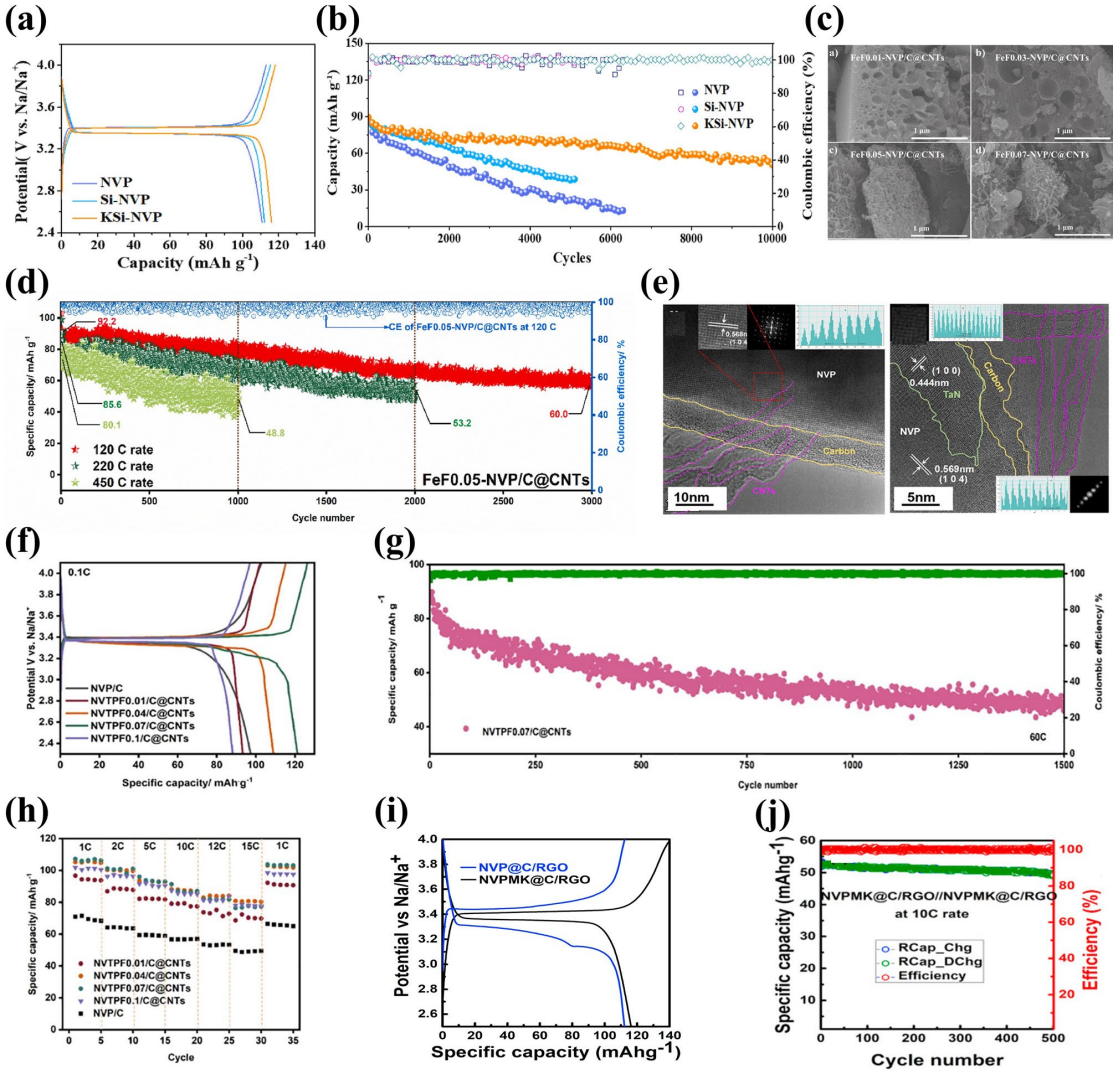
**a** Charge–discharge curves at 0.5 C and **b** cycling performance at 20 C of NVP, Si-NVP and KSi-NVP. Reproduced with permission [197]. Copyright 2023, Elsevier. **c** SEM images of FeF0.01-NVP/C@CNTs, FeF0.03-NVP/C@CNTs, FeF0.05-NVP/C@CNTs and FeF0.07-NVP/C@CNTs composites, **d** cycling performance of FeF0.05-NVP/C@CNTs at 120, 220 and 450 C. Reproduced with permission [198]. Copyright 2021, Elsevier. **e** HRTEM images of NVTPF0.07/C@CNTs materials, **f** charge–discharge curves of different NVTPF/C@CNTs cathodes, **g** cycling performance of NVTPF0.07/C@CNTs at 60 C, **h** rate capability of different NVTPF/C@CNTs cathodes. Reproduced with permission [188]. Copyright 2024, Elsevier. **i** Charge–discharge curve at 1 C, **j** cycling performance at 10 C of NVPMK@C/RGO//NVPMK@C/RGO. Reproduced with permission [115]. Copyright 2020, Elsevier

Multi-sites doping or substitution by virtue of the different doping sites in the NVP system, an optimization strategy combined with their independent benefits, has favorable contribution to the enhancement of electrochemical performance [196]. To substitute the Na and PO_4_^3−^ sites, Dou et al. [197] reported a Na_3.24_K_0.10_V_2.01_(PO_4_)_3_(SiO_4_)_0.14_ (KSi-NVP) cathode material. The introduction of SiO_4_^3−^ into PO_4_^3−^ site elevated the occupancy ration at Na2 site from 0.3489 to 0.4041, suggesting that more Na^+^ would participate electrochemical reaction to increase capacity. Meanwhile, the introduction of K^+^ into Na site as pillar ions lowed the occupancy ration at Na1 site from 0.1484 to 0.1176, which was of significance for the enhancement of structural stability in the KSi-NVP material. The optimized KSi-NVP exhibited a satisfactory discharge capacity of 116.3 mAh g^−1^ at 0.5 C, as well as ultra-long cycling life for 10,000 cycles at 20 C with merely 0.0056% capacity decay per cycle (Fig. 15a, b).

Besides, Chen et al. [198] proposed a Fe and F co-doped strategy by replacing the V and PO_4_^3−^ sites and synthesized a series of Na_3_V_2-*x*_Fe_*x*_(PO_4_)_3-*x*_F_3*x*_/C@CNTs (*x* = 0, 0.01, 0.03, 0.05, 0.07) materials. The doping of cationic Fe^3+^ not only enlarged intercellular voids but also facilitated the formation of porous structure (Fig. 15c), which greatly boosted the ionic conductivity and ameliorated the volume expansion/shrinkage. F^−^ was favor for reducing the particle size, which shortened the diffusion pathways of Na^+^ and further improved the ionic conductivity. Coupled with the highly conductive interconnected network by the combination of carbon coating and CNTs, ultimately, the Na_3_V_1.95_Fe_0.05_(PO_4_)_2.95_F_0.15_/C@CNTs harvested eye-catching high-rate capability and splendid long cycling stability, delivering a 92.2 mAh g^−1^ capacity at 120 C with 65.1% capacity retention after 3000 cycles and 80.1 mAh g^−1^ at 450 C (Fig. 15d). Subsequently, Chen et al. [188] selected Ta^5+^, having higher valence and larger size than V^3+^, to partially substitute V site and F^−^ to partially substitute PO_4_^3−^ site, and obtained Na_3_V_1.95_Ta_0.05_(PO_4_)_1.93_F_0.21_/C@CNTs (NVTPF0.07/C@CNTs) composites. The *n*-type effect of Ta^5+^ dopant effectively increased electronic conductivity by inducing more amounts of free electrons. The introduction of Ta^5+^ expanded the transportation pathways of Na^+^ and strengthened the structural stability of NVP system. Interestingly, a nascent conductive TaN phase incorporated with NVP bond formed a specific TaN/NVP heterojunction (Fig. 15e), significantly accelerating the Na^+^ diffusion. Benefiting from the synergistic effects of Ta and F co-doping, the NVTPF0.07/C@CNTs cathode material showed a reversible capacity of 121.5 mAh g^−1^ at 0.1 C, 63.49% capacity retention after 1500 cycles at 60 C and exceptional rate performance with 91.3 mAh g^−1^ specific capacity at 10 C (Fig. 15f-h). In addition, the replacement of Na and V sites by K^+^/Mg^2+^ co-doping was achieved by Das et al. [115]. The modified Na_2.91_K_0.09_V_2.93_Mg_0.07_(PO_4_)_3_@C/rGO (NVPMK@C/rGO) cathode material displayed a high discharge capacity of 116.6 mAh g^−1^ at 1 C (Fig. 15i). Together, the assembled symmetrical full cell by NVPMK@C/rGO electrodes sustained 95% capacity retention after 500 cycles at 10 C (Fig. 15j).

Based on the above discussion, it can be found that the foreign-ion doping or substitution in the Na, V and PO_4_^3−^ sites is a very effective optimization strategy to improve the electrochemical performance of NVP cathode material. Specifically, it is worthwhile to consider that some transition metal elements with the advantages of eco-friendliness, low-cost and enabling high electrochemical properties, such as Fe, Mn, Al, Mg and Ti, are responsible for the best metal ion's substitution in NVP. Moreover, F^−^ and SiO_4_^4−^ should be considered as the best anion doping in NVP due to the strong electronegative or identical ion structure with PO_4_^3−^. The doping or substitution in different sites exhibits various effectiveness for enhancing the sodium storage performance of NVP cathode material. Notably, the replacement of V site has been regarded as a more effective approach to boost the properties of NVP material compared with the doping or substitution in Na and PO_4_^3−^ sites, which includes isovalent-ion substitution, aliovalent-ion substitution and multiple-ions substitution, presenting different potentialities for optimizing the electrochemical performance of NVP material. Moreover, it is the multiple-ions doping or substitution in V site that can incorporate the respective advantages of every metal ion and achieve excellent electrochemical performance of NVP material. In addition, multi-sites doping or substitution, combining with the independent benefits of different doping sites, is also proven to be an effective strategy to enhance the electrochemical performance of NVP material. Table 2 compares the electrochemical performance of some foreign-ion doped NVP cathode materials introduced previously.

**Table 2:** Comparison of electrochemical performance of some reported foreign-ion doped NVP cathode materials

Site	Cathode material	Doping ion	Voltage range (V)	Discharge capacity (mAh g^−1^)/Rate	Capacity retention/Cycles/Rate	Refs
Na	Na_2.9_K_0.1_V_2_(PO_4_)_3_/C	K^+^	2.5–3.8	107.7, 0.2 C	95%, 300, 0.2 C	[67]
	Na_2.95_K_0.05_V_2_(PO_4_)_3_	K^+^	2.5–3.8	100.1, 0.5 C	80%, 1000, 10 C	[117]
	Na_2.8_Li_0.2_V_2_(PO_4_)_3_/C	Li^+^	2.5–3.8	116.9, 0.2 C	99.82%, 500, 0.2 C	[118]
	Na_2.8_Ca_0.1_V_2_(PO_4_)_3_@C-gC_3_N_4_	Ca^2+^	2.3–3.9	106.4, 0.1 C	93.6%, 3000, 10 C	[121]
V	Na_3_V_1.5_Cr_0.5_(PO_4_)_3_	Cr^3+^	1.0–4.4	150, 30 mA g^−1^	96%, 400, 30 mA g^−1^	[132]
	Na_3_V_1.5_Cr_0.5_(PO_4_)_3_	Cr^3+^	1.0–4.2	163.2, 0.1 C	87.5%, 1000, 1 C	[124]
	Na_3_Cr_4/3_V_3/2_(PO_4_)_3_@C	Cr^3+^	1.5–4.4	175, 100 mA g^−1^	83%, 2000, 100 mA g^−1^	[136]
	Na_3_Cr_0.5_V_1.5_(PO_4_)_3_ /C@rGO	Cr^3+^	1.0–4.4	176, 0.2 C	80.49%, 100, 0.2 C	[134]
	Na_3_V_1.6_Cr_0.4_(PO_4_)_3_	Cr^3+^	2.5–4.1	115, 0.1 C	95%, 100, 0.1 C	[135]
	Na_3_V_1.98_Al_0.02_(PO_4_)_3_/C	Al^3+^	2.3–3.8	102.7, 10 mA g^−1^	99.2%, 50, 10 mA g^−1^	[47]
	Na_3_V_1.5_Al_0.5_(PO_4_)_3_	Al^3+^	1.0–4.4	165, 0.1 C	87.9%, 500, 5 C	[142]
	Na_3_V_1.25_Ga_0.75_(PO_4_)_3_	Ga^3+^	1.4–4.2	152.3, 1 C	84.52%, 600, 10 C	[61]
	Na_3_V_1.25_Ga_0.75_(PO_4_)_3_	Ga^3+^	2.2–4.2	105, 1 C	92.3%, 400, 1 C	[61]
	Na_3_V1_.5_Mn_0.5_(PO_4_)_3_	Mn^3+^	1.0–4.0	170.9, 0.5 C	/	[129]
	Na_3_V_1.5_Fe_0.5_(PO_4_)_3_	Fe^3+^	1.0–4.5	166.4, 1 C	80.41%, 50, 1 C	[143]
	Na_3_Fe_0.8_V_1.2_(PO_4_)_3_/C	Fe^3+^	1.5–4.5	115.2, 0.5 C	97.8%, 500, 5 C	[62]
	Na_3_V_1.85_Fe_0.15_(PO_4_)_3_@C	Fe^3+^	2.3–4.3	103.9, 1 C	91.45%, 1200, 1 C	[131]
	Na_3_V_1.96_Ru_0.04_(PO_4_)_3_/C@CNTs	Ru^3+^	2.3–4.1	112.7, 1 C	86.63%, 14,800, 80 C	[73]
	Na_3_V_1.97_Bi_0.03_(PO_4_)_3_/C@CNTs	Bi^3+^	2.3–4.1	112.6, 0.1 C	82.41%, 9000, 12 C	[88]
	Na_3_V_1.93_Y_0.07_(PO_4_)_3_	Y^3+^	2.3–4.1	125.2, 0.1 C	85.5%, 2000, 250 C	[145]
	Na_3_V_1.8_Mn_0.2_(PO_4_)_3_/C	Mn^2+^	2.5–4.0	106.8, 1 C	82%, 10,000, 30 C	[149]
	Na_3.75_V_1.25_Mn_0.75_(PO_4_)_3_	Mn^2+^	2.5–4.1	100, 1 C	96%, 100, 5 C	[151]
	Na_3.5_Mn_0.5_V_1.5_(PO_4_)_3_	Mn^2+^	2.5–4.1	108.3, 2 C	87.2%, 4000, 20 C	[152]
	Na_3_V_5.92/3_Mn_0.04_(PO_4_)_3_/C@CNTs@ 1wt%Al_2_O_3_	Mn^2+^	2.3–4.1	115.9, 1 C	84.87%, 6000, 30 C	[99]
	Na_3.5_Mn_0.5_V_1.5_(PO_4_)_3_ @GO	Mn^2+^	2.5–4.2	112, 2 C	81.3%, 400, 2 C	[147]
	Na_3.4_V_1.6_Fe_0.4_(PO_4_)_3_	Fe^2+^	2.5–4.1	133, 0.5 C	96%, 2000, 20 C	[158]
	Na_3.5_V_1.5_Fe_0.5_(PO_4_)_3_	Fe^2+^	1.7–4.3 2.0–3.8	148.2, 0.5 C 112, 0.5 C	72%, 500, 5 C 92%, 1000, 5 C	[160]
	Na_3.01_V_1.99_Co_0.01_(PO_4_)_3_/C	Co^2+^	2.5–4.0	116, 0.5 C	83.2%, 1000, 10 C	[161]
	Na_3.03_V_1.97_Cu_0.03_(PO_4_)_3_/C	Cu^2+^	2.5–4.0	114, 0.5 C	93.1%, 1000, 10 C	[161]
	Na_3_V_1.93_Co_0.07_(PO_4_)_3_/C	Co^2+^	2.3–4.1	114.4, 0.1 C	80.51%, 1000, 10 C	[162]
	Na_3_V_1.95_Ca_0.05_(PO_4_)_3_/C	Ca^2+^	2.0–3.9	111.4, 1 C	90.4%, 300, 1 C	[163]
	Na_3_V_1.97_Ni_0.03_(PO_4_)_3_/C	Ni^2+^	2.0–3.8	108.2, 1 C	93.5%, 50, 5 C	[165]
	Na_3_V_1.95_Mg_0.05_(PO_4_)_3_/C	Mg^2+^	2.5–4.0	112.5, 1 C	81%, 50, 20 C	[166]
	Na_3.04_V_1.96_Mg_0.04_ (PO_4_)_3_/C	Mg^2+^	2.5–4.2	123.8, 1 C	89.1%, 3000, 20 C	[60]
	Na_3_V_1.95_Ca_0.05_(PO_4_)_3_@C	Ca^2+^	2.0–4.3	116, 1 C	93%, 1000, 1 C	[170]
	Na_3_V_1.965_Ca_0.04_(PO_4_)_3_@C @CNTs	Ca^2+^	2.3–4.1	117.4, 0.1 C	95%, 4000, 50 C	[171]
	Na_3_V_1.9_Ti_0.1_(PO_4_)_3_/C	Ti^4+^	2.0–4.2	123.3, 0.1 C	62.3%, 8000, 20 C	[174]
	Na_2.85_V_1.85_Ti_0.15_(PO_4_)_3_	Ti^4+^	2.3–3.9	101.5, 2 C	60%, 2000, 10 C	[175]
	Na_2.9_V_1.98_Mo_0.02_(PO_4_)_3_	Mo^6+^	2.2–3.8	112.5, 0.5 C	83.5%, 500, 10 C	[176]
	Na_3_Fe_0.8_VNi_0.2_(PO_4_)_3_	Fe^2+^, Ni^2+^	2.0–3.8	102, 0.1 C	84.6, 1400, 20 C	[140]
	Na_3_V_1.5_Cr_0.4_Fe_0.1_(PO_4_)_3_	Fe^2+^, Cr^3+^	2.5–4.3	114.7, 0.1 C	90%, 10,000, 30 C	[110]
	Na_4_FeV_1/3_Ti_2/3_(PO_4_)_3_	Fe^2+^, Ti^3+^	1.3–4.3	125.83, 0.1 C	92.8%, 500, 0.5 C	[177]
	Na_3.75_V_1.25_Mn_0.5_Ni_0.25_(PO_4_)_3_	Mn^2+^, Ni^2+^	2.0–4.0	108.1, 0.2 C	85.8%, 1000, 5 C	[111]
	Na_3.2_Mn Ti_0.8_ V_0.2_ (PO_4_)_3_	Mn^2+^, Ti^4+^	1.5–4.4	172.5, 50 mA g^−1^	84.1%, 100, 100 mA g^−1^	[125]
	Na_3.2_V_2.7_(FeMnCo)_0.1_(PO_4_)_3_	Fe^3+^, Mn^2+^, Co^2+^	2.3–4.1 2.0–4.5	122.2, 0.1 C 166.3, 1 C	88.1%, 800, 1 C 80.1%, 800, 1 C	[113]
	Na_3.5_Fe_0.5_V_0.5_Cr_0.5_Ti_0.5_(PO_4_)_3_	Fe^2+^, Cr^3+^, Ti^3+^	1.5–4.5	178.3, 0.1 C	69.48%, 5000, 5 C	[126]
	Na_3_VAl_0.2_Cr_0.2_Fe_0.2_In_0.2_Ga_0.2_(PO_4_)_3_	Al^3+^, Cr^3+^, Fe^3+^, In^3+^, Ga^3+^	2.5–4.4	102, 0.1 C	86.8%, 5000, 20 C	[180]
	Na_3.32_V_1.6_Cr_0.08_Fe_0.08_Mn_0.08_Mg_0.08_Ca_0.08_(PO_4_)_3_	Cr^3+^, Fe^2+^, Mn^2+^, Mg^2+^, Ca^2+^,	1.2–4.2	120, 5.0 A g^−1^	93.1%, 2000, 5.0 A g^−1^	[181]
	Na_3.4_Fe_0.4_Mn_0.4_V_0.4_Cr_0.4_Ti_0.4_(PO_4_)_3_	Fe^2+^, Mn^2+^, Cr^3+^, Ti^3+^	1.5–4.5	161.3, 0.1 C	85.3%, 1000, 5 C	[182]
PO_4_^3−^	Na_3_V_2_(PO_4_)_1.93_F_0.0_/C	F^−^	2.3–4.2	113, 10 mA g^−1^	86%, 1000, 200 mA g^−1^	[191]
	Na_3_V_1.98_(PO_4_)_2.9_F_0.3_/C	F^−^	2.3–4.2	116.9, 0.1 C	89.3%, 100, 1 C	[192]
	Na_3_V_2_(PO_4_)_3–1/6_(BO_3_)_1/6_	BO_3_^3−^	2.5–4.0	105, 1 C	98.7%, 300, 5 C	[185]
	Na_3.1_V_2_(PO_4_)_2.9_(SiO_4_)_0.1_	SiO_4_^2−^	2.3–3.9	109.4, 0.2 C	98%, 500, 1 C	[183]
	Na_3.1_V_2_(PO_4_)_2.9_(SiO_4_)_0.1_/C@rGO	SiO_4_^2−^	2.3–4.1	113.6, 0.1 C	76.7%, 2000, 6 C	[195]
	Na_3.2_V_2_(PO_4_)_2.9_(MoO_4_)_0.1_	MoO_4_^2−^	2.4–4.3	108.9, 1 C	91.5%, 150, 1 C	[187]
Multiple-site	Na_3.24_K_0.10_V_2.01_(PO_4_)_3_(SiO_4_)_0.14_	K^+^, SiO_4_^2−^	2.3–4.1	116.3, 0.5 C	44%, 10,000, 20 C	[197]
	Na_3_V_1.95_Fe_0.05_(PO_4_)_2.95_F_0.15_/C@CNTs	Fe^3+^, F^−^	2.3–4.1	111.1, 0.1 C	65.1%, 3000, 120 C	[198]
	Na_3_V_1.95_Ta_0.05_(PO_4_)_1.93_F_0.21_/C@CNTs	Ta^5+^, F^−^	2.3–4.1	121.5, 0.1 C	63.49%, 1500, 60 C	[188]
	Na_2.91_K_0.09_V_2.93_Mg_0.07_(PO_4_)_3_@C/rGO	K^+^, Mg^2+^	2.5–4.0	116.6, 1 C	/	[115]

### Nanostructure and Morphology Design

Different dimensional structures with unique morphologies of NVP cathode materials have been constructed and demonstrated as an effective way to enhance their rate capability and cycle stability [199–201]. Based on various kinds of preparation methods and nanotechnologies, three types of dimensional structures with notable morphologies can be designed for the NVP materials, such as 1D nanofibers/nanowires, 2D nanoflake/nanosheet/nanoplate and 3D nanosphere/hierarchical porous/hollow structures. The nano-designing endows NVP with many advantages. Firstly, the unique morphologies with larger specific surface area can promote electrolyte penetration and boost electrons transportation. Secondly, the nanostructure shortens Na^+^ ions diffusion distance and elevates ionic conductivity. Lastly, the nano-construction of NVP materials generates additional voids and cavities for the great alleviation of cell volume change and thus stabilizes the crystal structural of NVP. The electrochemical performance of some reported NVP cathode materials with different structures and morphologies is listed in Table 3.

**Table 3:** Electrochemical performance of some reported NVP cathode materials with different dimensional nanostructures and morphologies

Cathode material	Method	Structure/Morphology	Discharge capacity (mAh g^−1^)/Rate	Capacity retention/Cycles/Rate	Refs
Na_3_V_2_(PO_4_)_3_/C	Electrospinning	Nanofibers	113, 0.1 C	97%, 100, 0.05 C	[200]
Na_3_V_2_(PO_4_)_3_/C	Ionic liquid (IL)-assisted electrospinning	Nanofibrous mats	117.4, 0.1 C	89.07%, 400, 1 C	[201]
Na_3_V_2_(PO_4_)_3_/C	Self-sacrificed template method	Nanofibers	113, 1 C	95.9%,1000, 10 C	[203]
Na_3_V_2_(PO_4_)_3_/C	Hydrothermal and post calcination	Nanoplates	117, 0.2 C	82.6%, 10,000, 50 C	[204]
Na_3_V_2_(PO_4_)_3_/C	Soft-template	Nanoflakes	115.4, 0.5 C	78.5%, 50,000, 100 C	[205]
Na_3_V_2_(PO_4_)_3_/C	Microwave-assisted hydrothermal	Nanosheets	116.6, 0.2 C	78.31%, 1000, 100 C	[206]
Na_3_V_2_(PO_4_)_3_/C	Hydrothermal	Hierarchical porous microspheres	116.3, 0.5 C	91.9%, 1000, 5 C	[208]
Na_3_V_2_(PO_4_)_3_/C	Solvothermal	Core–shell micellar structure	115.2, 1 C	79.53%, 1000, 10 C	[202]
Na_3_V_2_(PO_4_)_3_/C	MOFs assisted sol–gel	Hierarchical porous micro-nano particles	110.4, 0.5 C	97.8%, 500, 1 C	[210]
Na_3_V_2_(PO_4_)_3_/C	Spray-drying	Micro-nano spherical	106, 2 C	50%, 5000, 50 C	[211]

**Fig. 16:**
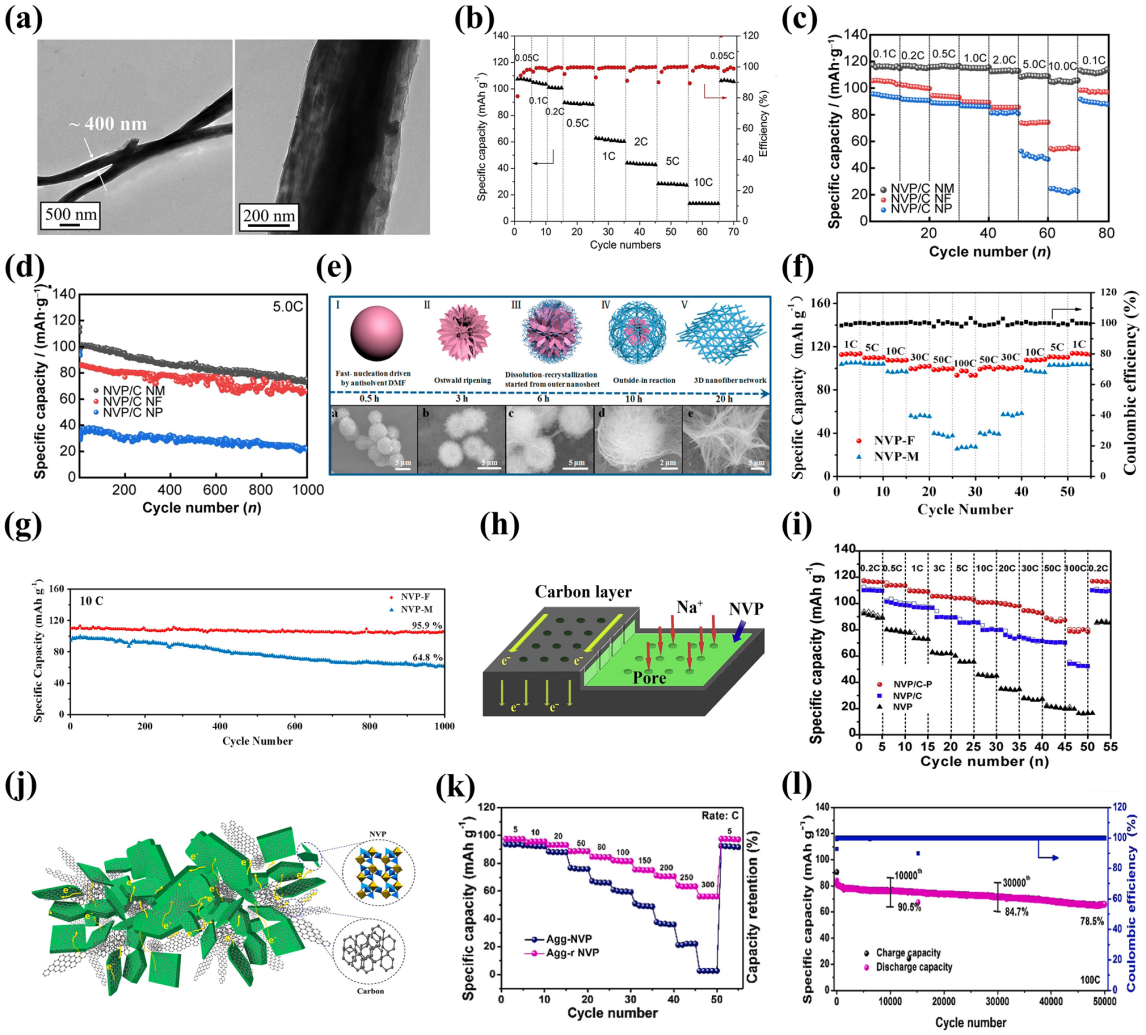
**a** TEM images of Na_3_V_2_(PO_4_)_3_/C sample with oxalic acid, **b** rate performances of Na_3_V_2_(PO_4_)_3_/C sample with oxalic acid. Reproduced with permission [200]. Copyright 2018, Elsevier. **c** Rate capability of NVP/C-NM and **d** cycling performance at 5.0 C of NVP/C-NM, NVP/C NF and NVP/C NP. Reproduced with permission [201]. Copyright 2021, Youke Publishing. **e** Schematic illustrations of the time-dependent solvothermal reaction: self-sacrificed evolution mechanism from microsphere to nanofiber network,** f** rate capability and **g** cycling performance of NVP-F and NVP-M electrodes. Reproduced with permission [203]. Copyright 2016, Elsevier. **h** Schematic illustration of constructions of NVP/C-P, **i** rate capability of NVP, NVP/C and NVP/C-P. Reproduced with permission [204]. Copyright 2018, Elsevier. **j** Schematic diagram of Agg-r NVP nanoflakes,** k** rate capability of Agg-NVP and Agg-r NVP, **l** cycling performance of Agg-r NVP electrode at 100  C. Reproduced with permission [205]. Copyright 2018, Elsevier

Electrospinning method, as a facile and environmental friendliness technique, is popularly employed to obtain 1D nanofiber materials with good properties. Wu et al. [200] synthesized Na_3_V_2_(PO_4_)_3_/C (NVP/C) nanofibers with smooth and uniform surface morphologies through electrospinning method with choosing oxalic acid as reductant, complexing agent and carbon source. As shown in Fig. 16a, the optimized 1D NVP/C nanofibers, which were characterized by homogenous and smooth surface and mean diameter of approximately 400 nm without any protrusions, could be witnessed. As a result, the modified NVP/C nanofibers cathode showed a good discharge capacity of 113 mAh g^−1^ at 0.1 C and better rate performance (Fig. 16b). Liu et al. [201] designed cross-welded Na_3_V_2_(PO_4_)_3_/C nanofibrous mats (NVP/C-NM) via electrospinning route, which selected ionic liquid as phosphoric acid source and carbon source to make sure the spinnability in the PAN-based electrospinning system and form a compact and continuous carbon layer. The NVP/C-NM served as a current collector and had a helpful impact on enlarging contact area between electrode and electrolyte, resulting in higher electrochemical performance compared with NVP/C nanofibers and NVP/C nanoparticles. As displayed in Fig. 16c, d, the NVP/C-NM cathode presented impressive rate capability and stable cycling performance. Besides, the structure and morphology of NVP nanomaterials can be regulated by introducing a template as guide, such as N-dimethylformamide (DMF), cetyltrimethylammonium bromide (CTAB), polyethylene glycol (PEG) and metal–organic frameworks (MOFs), which can obtain different nano-dimensional NVP materials [87, 202]. Mai’s group [203] proposed a simple self-sacrificed template method and successfully synthesized 1D NVP nanofibers (NVP-F). By adding DMF and controlling reaction time (from 0.5 to 20 h), the resultant 1D NVP nanofibers with diameters of 20–80 nm and length of about millimeters were observed, which evolved from microsphere to 3D nanofiber network (Fig. 16e). Importantly, it was 3D nanofiber network that generated more gaps for providing more Na^+^ ion-transport channels and enlarged specific surface area for sufficient penetration of electrolyte. Consequently, the NVP-F harvested superior cycling stability, sustaining 95.9% capacity over 1000 cycles at 10 C, together with excellent high-rate performance (94 mAh g^−1^ at 100 C) (Fig. 16f, g).

The 2D NVP nanoplates (NVP/C-P) with abundant pores effectively shortened Na^+^ ions diffusion paths and enlarged specific surface area (Fig. 16h) [204]. Compared to the bare NVP and NVP/C particles, the NVP/C-P cathode material delivered a higher discharge capacity of 117 mAh g^−1^ at 0.2 C, and exhibited superior rate capability (87.3 mAh g^−1^ specific capacity at 50 C) (Fig. 16i) and outstanding cycling stability (82.6% capacity retention at 50 C over 10,000 cycles). Guo et al. [205] skillfully synthesized 2D Na_3_V_2_(PO_4_)_3_ (NVP) nanoflakes with plane dimensional sizes of 100 to 150 nm, drawing support from the soft template of ethylene glycol (EG). The NVP nanoflakes could promote crystal nucleation and subsequently transform into super electronic network in the heat treatment. As depicted in Fig. 16j, the NVP nanoflakes were homogeneously dispersed in the electronic network and effectively mitigated the restacking problem of 2D nanoflakes. Meantime, the existence of massive interstitial voids between nanoplates elevated rapid charge transfer across the electrode/electrolyte interface. The NVP nanoflakes exhibited ultrahigh-rate performance with about 71.2 and 56.2 mAh g^−1^ discharge capacities at 200 and 300 C, respectively (Fig. 16k). Astonishingly, the optimized NVP yielded a high initial specific capacity of 84.1 mAh g^−1^ at 100 C with 78.5% capacity retention after 50,000 cycles (Fig. 16l).

**Fig. 17:**
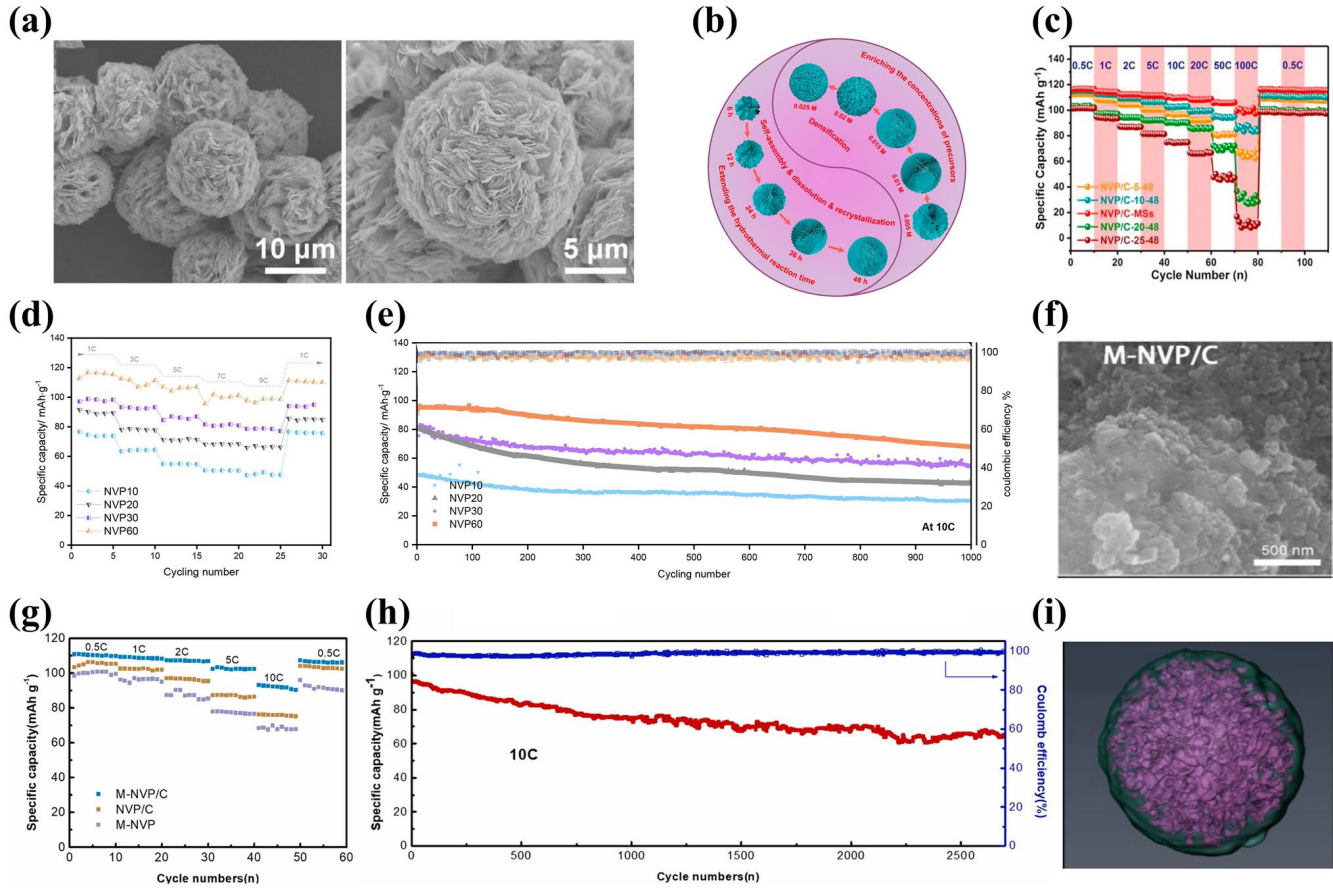
**a** SEM images of NVP/C-MSs, **b** schematic illustration of formation process for hierarchical microspheres, **c** rate capability of different NVP/C microspheres. Reproduced with permission [208]. Copyright 2019, Elsevier. **d** Rate performance and **e** cycling performance of NVP cathodes synthesized at different conditions. Reproduced with permission [202]. Copyright 2022, Elsevier. **f** SEM image of M-NVP/C sample, **g** rate capability of M-NVP, NVP/C and M-NVP/C samples, **h** cycling performance of M-NVP/C at 10 C. Reproduced with permission [210]. Copyright 2022, Elsevier. **i** 3D model of NVP particle. Reproduced with permission [211]. Copyright 2019, Elsevier

However, the low-dimensional structures of nanomaterials (nanofibers, nanoplates and nanoflakes) are prone to agglomerate during the high-temperature treatment and repetitive charging/discharging processes, resulting in the loss of active materials and inferior sodium storage performance [206]. To deal with this issue, some researches ingeniously employed low-dimensional nanomaterials to construct 3D hierarchical porous architectures, which could not only significantly suppress the agglomeration of NVP nanomaterials but also further reduce the transmission distance of Na^+^ ions and electrons [207–209]. Cao et al. [208] used a hydrothermal method to synthesize 3D Na_3_V_2_(PO_4_)_3_/C hierarchical microspheres (NVP-MSs) assembled from loosely interconnected 2D nanoflakes, which had the thickness of about 20–30 nm and existed distinct open space between them (Fig. 17a). As shown in Fig. 17b, the morphological evolution of NVP hierarchical microspheres could be controlled by tuning the precursor concentration and hydrothermal reaction time. The optimized NVP-MSs exhibited a nearly theoretical capacity of 116.3 mAh g^−1^ at 0.5 C, as well as a desirable discharge capacity of 99.3 mAh g^−1^ at 100 C (Fig. 17c).

Furthermore, some novel optimization strategies for constructing 3D structures with unique shapes of NVP are proposed, without the help of low-dimensional nanomaterials [202, 210]. Sun et al. [202] constructed 3D crosslinked core–shell micellar structures and prepared nanoflowers-shaped NVP/C materials combined with negative flake V_2_O_5_ by adding structural guiding agent PEG and surfactant CTAB in the 100% methyl alcohol solvent system. Specifically, the PEG and CTAB played an effective synergistic effect on the formation of the unique nanoflower-shaped NVP (positive charge). Meanwhile, the low polarity methanol protected the nanoflowers from breaking original morphologies. On accountant of the charge attraction, the negative platelike V_2_O_5_ was compactly absorbed on the surface of NVP, contributing to more active sites for NVP materials. Benefiting from these merits, the optimized NVP cathode obtained outstanding rate performance and cyclic stability, delivering a reversibility capacity of 115.2 mAh g^−1^ at 1 C and 98.7 mAh g^−1^ at 10 C after 1000 cycles (Fig. 17d, e).

In addition, Chen et al. [210] prepared micro-nano NVP/C materials (M-NVP/C) with micro-size hierarchical porous structure and nano particles (Fig. 17f), derived from MOFs precursors. MOFs materials acting as a guider had a positive effect to construct hierarchical porous structure, which possessed high specific surface area to allow ample infiltration of electrolyte and effectively restricted agglomeration of nanoparticles. As displayed in Fig. 17g, h, the M-NVP/V submitted a remarkable discharge capacity of 95.0 mAh g^−1^ at 10 C and 67.7% capacity retention after 2700 cycles at 10 C. Moreover, Yang et al. [211] also developed 3D micro-nano spherical structure NVP/C materials (Fig. 17i) composed of micro spherical particles and internal nano channels via spray-drying method. The as-prepared NVP/C with micro-size displayed competitive rate capability with 106, 104, 101, 96, 89, 70 and 44 mAh g^−1^ discharge capacities at 2, 5, 10, 20, 50, 100 and 200 C, respectively, compared to the nano sized NVP/C with 106, 103, 85, 77 and 60 mAh g^−1^ discharge capacities at 2, 5, 10, 20 and 50 C, respectively.

Generally, the optimal design of structures and morphologies of NVP nanomaterials plays a significant role to optimize the electrochemical performance of NVP cathode materials. Nevertheless, there remain some challenges. Firstly, it is the high specific surface area that is frequently produced through high carbon contents, reducing the volume energy density. Besides, nanoparticles are easily agglomerating and taking side reactions during the high-temperature sintering process and subsequent electrochemical cycles, which limit the utilization of active materials and cause unsatisfactory electrochemical properties. Conversely, the nanomaterials, in the low temperature, obtain inferior crystallinity and impurity phases, which influence the further research. Thirdly, the synthesis routes of NVP nanomaterials are usually complex and adverse to achieve large-scale applications in SIBs.

## Summary and Outlook

As a promising cathode material for SIBs, Na_3_V_2_(PO_4_)_3_ (NVP) has been a research hotpot in terms of its robust structure with sufficient ion-diffusion channels and good thermal stability. Nevertheless, NVP remains challenging for the achievement of high capacity and satisfactory energy density because of its poor electronic conductivity and sluggish diffusion kinetics. Currently, significant endeavors to optimize NVP cathode material have been witnessed. Many optimization strategies have been proposed to optimize the electrochemical performance of NVP cathode material. In this review, we have completely summarized and discussed the latest advances in the optimization strategies of NVP cathode material, including carbon coating or modification, foreign-ion doping or substitution and nanostructure and morphology design. The carbon coating or modification, such as coating carbon layer on the surface of NVP or preparing NVP/carbon composite, is a preferred strategy to boost electronic conductivity and suppress the adverse agglomeration of NVP particles. The foreign-ion substitution or doping into NVP, occurring in the Na, V and PO_4_^3−^ sites, has become a primary research trend to intrinsically improve its electrical conductivity. The doping or substitution includes single-site doping and multiple-site doping, and for single-site doping, there are single-ion doping and multiple-ion doping. The doping or substitution in V site is very effective for enhancing the sodium storage performance of NVP. It is of constructive significance to rationally design nanostructure with specific morphology comprising 1D, 2D or 3D architectures, which enable NVP with fast Na ion-diffusion kinetics and substantial interfacial contact with electrolyte. Based on these optimization strategies, the electrochemical performance of NVP cathode has been greatly improved, such as its reversible capacity, rate capability and cycling stability.

However, every optimization approach still has some weaknesses. Although carbon materials loaded on NVP can increase electronic conductivity efficiently, most of carbon coating and composing with high carbon content may slow down the transfer of electrons and ions and impair the capacity of NVP. The alien ions introduced into NVP will inevitably give rise to crystal distortion and thus lead to unsatisfactory cycling stability in view of the lattice compatibility. In addition, complex preparation and technologies of nanostructured NVP are hardly to meet with the large-scale and low-cost commercial requirements for the practical applications. Meantime, nanosized NVP particles easily agglomerate and therefore decrease the availability of NVP cathode materials. Accordingly, aiming at the high-performance NVP cathode materials in the field of commercialization for SIBs, to further develop new optimization strategies is imperative. Herein, we propose some considerations and perspectives for the further explorations in designing and developing high-performance NVP cathode materials for practical application in SIBs.

### Optimizing Carbon Coating/Modification

Carbon-based materials have a great influence on the electrochemical performance of carbon-modified NVP cathodes. Selecting suitable carbon source is very important for improving performance and reducing cost. The biomass-derived carbon featuring low-cost, adjustable structure and various heteroatom doping can be a suitable alternative as carbon source to optimize NVP materials. Besides, constructing NVP@C@C 3D spatial structure with combining carbon composite and carbon coating can realize rapid transfer and diffusion of electrons and ions, which is helpful to improve rate capability and cycling performance. Moreover, low loading content and appropriate thickness of carbon layer coated on NVP are critical to efficiently boost electronic conductivity and improve electrochemical activity of NVP. New strategies should be developed to construct NVP/C composites with lower carbon loading content and appropriate thickness of carbon layer. The construction of carbon dot structure is worthy of concentration and necessary to be further investigated. The carbon dot incorporation strategy with trace carbon content can not only significantly boost the electronic conductivity but also elevate the electrochemical activity of NVP [96].

### Integrating and Optimizing Multiple-Ion and Multi-Site Substitution

Foreign-ion doping or substitution is very effective for enhancing electrical conductivity and improving electrochemical performance of NVP cathodes. Most investigations of foreign-ion doping are mainly focused on the single-ion doping at V site. Multiple-ion and multi-site doping or substitution can well combine their own advantages to induce synergistic effect for further improving the electrochemical performance of NVP cathodes, which should be further investigated. These co-substitution strategies of different substitution elements functioned at different sites will be greatly beneficial for the enhancement of rate capability and long-term cyclic stability for NVP cathodes. Furthermore, the concept of high-entropy as a new insight has been employed to optimize the crystal structure of NVP cathode material for enhancing its electrochemical performance. The optimized mechanism and composition of high-entropy modified NVP needs to be explored in depth. In addition, considering the practical application, the V content in NVP need be reduced due to its high cost and toxicity. The low-cost, safe and earth-abundant elements, such as Mn, Fe and Ni, should be selected as transition metal elements for the substitution of V in NVP. The high throughput screening method should be considered for greatly facilitating the investigation to find new composition and structures.

### Optimizing Nanostructure Construction

Nanoscale structure of NVP cathode material exerts positive impacts for narrowing the Na ion-migration pathways, enlarging electrode/electrolyte contact area and accelerating the transfer of ion. Constructing reasonable nanostructured NVP cathode material will contribute to high electrochemical performance. Much efforts need to be made to design and develop nanostructured NVP materials. Moreover, the nanostructure design should be combined with carbon coating or modification to construct 3D conducting network of NVP, which will help to increase specific capacity, enhance rate capability and improve cycling stability. However, the synthesis processes of NVP nanocomposites are generally complicated and time-consuming, which restricts their practical applications. Therefore, it is an essential thing to develop facile and efficient preparation route to construct nanostructured NVP cathode materials.

### Choosing Suitable Electrolyte and Optimizing Cathode/Electrolyte Interface

Electrolyte is also an important component in battery, which affects the electrochemical performance of battery. Choosing suitable electrolyte will be beneficial to enhance the electrochemical performance of NVP cathode. The composition, including sodium salts, solvents and additives and concentration of electrolyte should be investigated for exploring their effects on the rate capability and cycling stability of NVP cathode. Besides, the electrode/electrolyte interface has also great influence on the electrochemical performance of NVP cathode. Constructing high-quality cathode/electrolyte interface can boost charge transfer kinetics and structural evolution of NVP cathode. Meantime, a compatible electrolyte can also promote the formation of a thin and uniform cathode/electrolyte interface. Therefore, the optimization of cathode/electrolyte interface plays a critical role to improve diffusion kinetics and increase electronic conductivity, which can reduce the efforts in different optimizing strategies to achieve high-performance NVP cathode material.

In summary, to achieve high-performance NVP cathode materials, focused efforts should be devoted to developing new optimization strategies. In addition, some proposed optimization strategies should be combined together to form synergistic effect for enhancing the electrochemical performance of NVP cathode materials. Though the commercialization of high-performance NVP cathode materials is a blocked and long road, with determined efforts, it will be finally achieved in the future.
